# Sexual dimorphism in cancer: molecular mechanisms and precision oncology perspectives

**DOI:** 10.1186/s13293-026-00843-7

**Published:** 2026-02-03

**Authors:** Zhen Wang, Hanwen Hu, Yunjia Bao, Guohong Ren, Chenghui Yang

**Affiliations:** 1 Key Laboratory of Tumor Microenvironment and Immune Therapy of Zhejiang Province, Second Affiliated Hospital, Zhejiang University School of Medicine, Hangzhou, 310009 P.R. China; 2 Department of Breast Surgery, Second Affiliated Hospital, Zhejiang University School of Medicine, Hangzhou, 310009 P.R. China; 3 First Clinical College of Wenzhou Medical University, Wenzhou, 325000 P.R. China; 4 Department of Breast Surgery, First Affiliated Hospital of Wenzhou Medical University, Wenzhou, 325000 P.R. China

**Keywords:** Sex differences, Cancer, Sex chromosomes, Sex hormones, Tumor microenvironment

## Abstract

Sex differences play a crucial role in determining tumor incidence, treatment sensitivity, and prognosis among men and women. However, current clinical cancer treatment strategies fail to account for these differences. Furthermore, the underlying mechanisms of tumor disparities between sexes remain elusive. Sex differences in sex chromosomes, hormone levels, metabolism, and immunity synergistically contribute to tumor-related disparities. As the demand for precision medicine escalates, there is an urgent need to conduct further exploration and research to address the tumor differences between sexes. In this review, we discuss the impact of biological sex differences on tumor cells and the tumor microenvironment, aiming to identify more effective strategies for tumor prevention and treatment.

## Introduction

Malignant tumors are major global public health problems that are serious threats to human quality of life and health. Epidemiological data show that there are significant sex differences in the incidence, progression, treatment response, and prognosis of cancer, but the underlying mechanism is not yet clear. In addition to the apparent presence of this sex difference in reproductive organ cancers, it is also observed in non-reproductive cancers. Although epidemiological sex disparities in cancer are associated with gender-related factors—including lifestyle, behaviors, and social roles, which are acquired and modifiable—we focus more on biological sex differences. These biological differences encompass genetic and physiological traits such as sex chromosome composition, sex hormone levels, and other phenotypic features including body structure, metabolic patterns, and immune function. In this review, we mainly discuss the effect of sex differences on tumors and summarize the factors and mechanisms through which sex differences lead to cancer differences. Understanding the intrinsic causes of sex differences in malignant tumors will provide new ideas for future tumor therapy based on sex differences.

## Epidemiological differences in cancer between different sexes

### Differences in cancer incidence

According to the latest statistics from the National Cancer Institute (NCI), an estimated 2,041,910 new cancer cases will be diagnosed in the United States in 2025, with persistent sex differences in cancer type, incidence, and mortality between males and females [1]. These differences are most prominently reflected in reproductive organ cancers—a congenital distinction rooted in biological sex. For males, prostate cancer is the most prevalent (accounting for 30% of all new male diagnoses), followed by lung and bronchial cancer (11%) and colorectal cancer (CRC, 8%); collectively, these three cancers represent 49% of all new male cases. In contrast, breast cancer dominates among females (32% of all new female diagnoses), with lung and bronchial cancer (12%) and CRC (7%) ranking second and third, together comprising 51% of new female cases [1].

Breast and prostate cancers are sex-specific by definition; when comparing non–sex-specific cancers, males show a consistently higher age-adjusted incidence rate. Data from the Surveillance, Epidemiology, and End Results (SEER) database provide additional corroboration of these long-term trends. Between 2001 and 2019, males consistently exhibited a higher age-adjusted incidence rate of lung cancer [2]. For CRC, the age-adjusted incidence rate in males remained 1.4-fold higher than that in females [3]. Besides, a recent SEER-based analysis of 5,000 CRC patients provides sex-stratified data that corroborate a male predominance in CRC incidence and highlights clinically relevant differences by sex across tumor sidedness, stage at diagnosis, treatment utilization, and survival. Consistent with our framework, the study reports sex-associated patterns in incidence and age at diagnosis (male-biased burden and distinct age distributions), Tumor location (right- vs. left-sided disease), Stage distribution and stage-specific outcomes, Utilization of systemic therapies and response metrics [4]. Even more pronounced disparities are observed in other non-reproductive cancers: male esophageal cancer incidence is 4-fold higher than that of females, and male kidney cancer incidence is 2-fold higher than that of females [5], confirming that sex disparities extend far beyond reproductive tissues.

A prospective cohort analysis from North America identified multiple factors potentially contributing to these sex differences, including risk behaviors (e.g., smoking, alcohol consumption), body structure, lifestyle factors, body mass index (BMI), and drug use (e.g., ibuprofen, aspirin). However, these factors only explained a small portion of the higher cancer risk in males across shared anatomical sites—sex differences persisted significantly even after adjusting for these variables. This male predominance underscores the critical role of sex-related biological factors in driving cancer incidence disparities [6].

### Differences in cancer prognosis

In addition to cancer incidence, sex differences also extend to cancer prognosis. Cancer mortality in men is almost twice as high as that in women [7]. Epidemiological data from the United States show that the highest number of cancer deaths in men are estimated to be lung cancer, prostate cancer, and CRC, while lung cancer, breast cancer, and CRC are estimated to occur in women [1].

A retrospective analytical study from Korean showed that male patients with non-small cell lung cancer (NSCLC) have a worse prognosis than female patients, even after adjusting for confounding clinical factors [8]. Notably, cc the age-adjusted death rate of CRC patients is approximately 1.5-fold greater in males than in females, and this difference becomes apparent after age 50 [3]. Similarly, a retrospective study of cutaneous malignant melanoma from Italy revealed women had better overall survival than men after adjustment for clinical factors [9].

A recent oncology cohort analysis of 1,726 adults with acute myeloid leukemia (AML) further highlighted sex-related prognostic differences: women were more frequently classified into the intermediate-risk group, whereas a higher proportion of men were categorized as adverse-risk [10, 11]. The significant difference in mortality between men and women is related to a variety of factors, including differences in chromosomal genes, circulating sex hormones, gut microbiota, and lifestyle factors. (Table 1)

**Table 1:** Incidence and prognosis of different cancer type between sex

No.	Year	Country	Enrolled Case Number or Database	Cancer Type	Incidence Rate (Male vs. Female)	Main Outcome	Results (Male vs. Female)	Ref.
1	2022	Italy	Male 678, Female 601	Cutaneous Malignant Melanoma	18.21 (16.76–19.76) vs. 17.66 (16.15–19.27).	OS; HR	48-month OS rate: 85.5% vs. 89.9%, *p* = 0.03; Overall survival rate (HR:1.41; 95% CI:1.03–1.93).	[9]
2	2022	Korea	Male 5943, Female 2707	non-small-cell lung cancer	N/A	Median survival; HR	Survival rate (HR:1.827; 95% CI:1.715–1.946, *p* < 0.001).	[8]
3	2022	United States	Male 294,998, Female 262,557	Lung cancer	N/A	HR	Survival specific deaths in women (HR: 0.83,95% CI: 0.82–0.83).	[12]
4	2019	Global	Data from IARC	Colorectal cancer	Incidence ASR (per 100,000 per year): 23.6 vs.16.3.	Mortality	Age-adjusted mortality (per 100,000 per year): 10.8 vs. 7.2.	[3]
5	2020	Global	Data from GLOBOCAN 2018 database	Bladder cancer	Incidence ASR (per 100,000): 9.6 vs. 2.4.	Mortality	The global ASR for mortality (per 100,000 per year): 3.2 vs. 0.9.	[13]
6	2021	Global	Data from 95 countries	Hepatocellular carcinoma	Incidence ASR (per 100,000): 11.6 vs. 3.4.	N/A	N/A	[14]
7	2017	United States	Data from SEER	Liver and Intrahepatic Bile Duct Cancer	Incidence (per 100,000): 11.8 (11.8–11.9) vs. 4.0 (4.0–4.1.0.1).	Mortality	Death rate (per 100,000): 9.2 (9.2–9.3) vs. 3.7 (3.7–3.8); 5-year survival rate: 20.9 (20.3–21.5) vs. 21.3 (20.3–22.3).	[15]
8	2023	United States	Data from SEER and the US Cancer Statistics database	Kidney and renal pelvis cancer	Incidence (per 100,000): 24.0 (23.8–24.1) vs.12.1 (12.0–12.2.0.2).	Mortality	Death rate (per 100,000): 5.1 (5.1–5.2) vs. 2.2 (2.2–2.2).	[16]
9	2015	Korea	Data from KCCR	Gastric cancer	Incidence (per 100,000): 99.89 (99.44–100.34.44.34) vs. 40.06 (39.79–40.32).	Mortality	Death rate (per 100,000 person-years): 52.75 (52.51–52.98) vs. 21.63 (21.50–21.77.50.77).	[17]
10	2022	China	Data from cancer registries in China	Gastric and esophageal cancer	Incidence ASR (per 100,000): 36.05 vs. 13.89.	Mortality	Mortality (1/100,000): 25.54 vs. 9.42.	[18]
11	2024	United States	Male 977, Female 749	Acute Myeloid Leukemia	N/A	OS, DFS	**CALGB/Alliance Cohort** Median OS (years): 1.5 vs. 1.9 Alive at 5 y, %: 35 (31–39) vs. 38 (33–43), *p* = 0.48 Median DFS (years): 1.7 vs. 1.6 Alive at 5 y, %: 39 (34–44) vs. 40 (34–45), *p* = 0.91.	[11]
12	2024	Germany	Male 489, Female 465	Acute Myeloid Leukemia	N/A	OS, DFS	**AMLCG Cohort** Median OS (years): 1.8 vs. 8.8; Alive at 5 y, %: 42 (36–48) vs. 51 (45–57), *p* = 0.005; Median DFS (years): 2.2 vs. 5.0; Alive at 5 y, %: 46 (38–54) vs. 51 (43–57), *p* = 0.17.	[11]

HR, Hazard Ratio; OS, Overall Survival; DFS, Disease-Free Survival; ASR, Age-standardized rate; KCCR, Korea Central Cancer Registry; SEER, The Surveillance, Epidemiology, and End Results; IARC, The International Agency for Research on Cancer

## Sex chromosome/genetic differences underlie sex disparities in cancer

Sex chromosome composition and genetic differences between males (XY) and females (XX) are inherent, foundational drivers of sex disparities in cancer incidence and progression [19]. The divergent expression of sex chromosome-specific genes—coupled with the male-specific presence of the Y chromosome and unequal X chromosome dosage between sexes—leads to abnormal chromosomal activation, inactivation, or gene expression, which further exacerbates sex-biased cancer development [20].

### X chromosome dysfunction

X chromosome inactivation (XCI) is a female-specific epigenetic process whereby one X chromosome is epigenetically “silenced” in most female somatic cells to avoid cytotoxicity from biallelic X-linked gene expression. However, if key tumor-related genes on the X chromosome escape XCI or if XCI maintenance is disrupted, it may either promote tumorigenesis or weaken tumor-suppressive effects. Notably, not all X-linked genes are fully inactivated: approximately 12–15% of X-linked genes can escape XCI, a phenomenon that protects females from complete functional loss of these genes due to single mutations [21, 22]. Additionally, multiple regions of the X chromosome harbor potential tumor suppressor genes that can escape XCI [23]. This may explain why females exhibit lower incidence and mortality for certain cancers compared to males [23].

The X inactive-specific transcript (*Xist*)—a gene expressed on the inactivated X chromosome—transcribes long noncoding RNAs (lncRNAs) to mediate chromosomal silencing. *Xist* plays a pivotal role in XCI establishment and maintenance, as it regulates epigenetic, transcriptional, and posttranscriptional processes with the help of the RNA-binding protein SPEN to stabilize XCI [24]. Abnormal *Xist* expression is closely associated with tumor progression. In human mammary stem cells (MaSCs), deletion of *Xist* reactivates genes on the previously silenced X chromosome; specifically, *Xist* drives enhancer overexpression in MaSCs via hyperactivation of MED14 (a subunit of the mediator complex), disrupting transcriptional programs and inducing abnormal MaSC differentiation—ultimately leading to breast cell transformation and poor prognosis in breast cancer patients [25]. In CRC, loss of METTL14 reduces the N^6^-methyladenosine modification of *Xist*, increasing its expression to promote tumor proliferation, invasion, and metastasis [26]. This *Xist*-mediated effect may contribute to male CRC susceptibility, as males—unlike females—possess only one X chromosome, rendering them more vulnerable to *Xist*-driven transcriptional disruption and subsequent tumor progression.

### Y chromosome dysfunction

As a male-specific chromosome, the expression of Y-linked genes or loss of the Y chromosome (LOY) directly modulates tumor cell function and immune evasion, contributing to male-predominant cancer aggressiveness.

#### Y Chromosome-specific oncogenes

Several Y-linked genes act as key drivers of male-specific tumor progression. In prostate cancer (PCa), the Y-linked long noncoding RNA *TTTY15* functions as a competing endogenous RNA by sponging microRNA let-7 to upregulate the expression of fibronectin 1 (FN1) and cyclin-dependent kinase 6 (CDK6), thereby promoting PCa cell migration and proliferation [27]. In hepatocellular carcinoma (HCC), the Y-linked proto-oncogene TSPY (testis-specific protein Y-encoded) is specifically expressed in a subset of male patients. TSPY upregulates cell cycle-related genes (e.g., *CDC25B*, *HMMR*) and growth factor receptor genes while inhibiting the tumor suppressor TP53, accelerating tumor cell proliferation and worsening prognosis in male HCC patients [28].

The Y-linked histone demethylase KDM5D exhibits context-dependent pro-tumor effects. In CRC mouse models, the *KRAS*-*STAT4* signaling pathway mediates KDM5D upregulation [29]. KDM5D epigenetically represses tight junction genes to disrupt cancer cell adhesion and inhibits MHC-I-mediated antigen presentation, suppressing CD8^+^ T cell antitumor immunity and driving invasion and metastasis [29].

#### Loss of the Y chromosome (LOY)

LOY is a common somatic mutation in males—prevalent in the peripheral blood of elderly men—and is strongly associated with cancer development. Analyses of Y chromosome genomes and cancer atlases show that extreme downregulation of Y-linked genes correlates with cancer status. Loss of six key Y-linked genes (*DDX3Y*, *EIF1AY*, *KDM5D*, *RPS4Y1*, *UTY*, *ZFY*) enhances cancer risk across cell types, as these genes regulate the cell cycle through distinct mechanisms [30]. In LOY mouse bladder cancer models, LOY tumors create an immunosuppressive tumor microenvironment (TME) characterized by increased tumor-associated macrophages, upregulated PD-1/PD-L1 expression, and CD8^+^ T cell exhaustion—facilitating tumor immune escape and growth [31]. Collectively, Y-linked oncogene activation and LOY synergistically explain why males exhibit higher CRC aggressiveness and worse prognosis than females.

Understanding how sex chromosome-linked genes modulate tumor cell biology and the tumor microenvironment can guide the development of sex-specific genetic or epigenetic biomarkers, improve risk stratification, and tailor targeted therapies such as inhibitors of X-linked oncogenes or Y-linked tumor suppressors.

## Sex-specific epigenetic regulation in cancer

Epigenetic modifications—heritable changes in gene expression occurring without alterations to the DNA sequence—are governed by three core mechanisms: DNA methylation, histone modifications, and non-coding RNA (ncRNA) regulation. Sex differences in cancer incidence, progression, and therapeutic response are increasingly recognized to be influenced by epigenetic regulation. Pervasive sex-biased patterns in DNA methylation and histone modifications across both normal tissues and malignant contexts. For instance, in a meta-analysis of 8,438 newborns, sex was associated with differential methylation at 46,979 autosomal CpG sites, with the majority of these sites exhibiting lower methylation levels in males than in females [32]. These differentially methylated sites are enriched in genes associated with cancer, psychiatric disorders, and cardiovascular phenotypes, suggesting that early-life epigenetic programming contributes to adult disease risk. Importantly, these epigenetic differences are not merely passive reflections of sex hormone levels, but are actively established and maintained through coordinated actions of epigenetic writers, erasers, and readers that integrate hormonal signals with sex chromosome–linked regulatory programs. In this section, we summarize current evidence supporting sex-specific epigenetic mechanisms in cancer, with a particular focus on how epigenetic machinery shapes sex-biased transcriptional programs and tumor phenotypes.

### Sex-biased epigenetic writers and erasers in cancer

#### DNA methylation machinery and sex differences

DNA methylation is the most extensively characterized epigenetic mark in the context of sexual dimorphism. As one of the most stable epigenetic marks contributing to long-term transcriptional control, DNA methylation displays a cancer landscape that differs markedly between males and females. In colorectal cancer, hypermethylation of X-linked genes including *FAM156B*, *PIH1D3*, and *PPP1R3F* has been linked to elevated colorectal cancer susceptibility, particularly with stronger associations observed in females [33]. Similarly, the functional inactivation of *FHL1*—the first identified fragile tumor suppressor on the X chromosome —via promoter methylation contributes to HCC progression, particularly in female liver cancer [34]. Conversely, in bladder cancer, male-predominant hypomethylation of the Y chromosome has been implicated in the higher incidence of disease in men [35].

Beyond X- and Y-linked events, autosomal DNA methylation has likewise been shown to exhibit substantial sex bias. A TCGA pan-cancer analysis of nine non-reproductive tumors identified thousands of CpG sites with attenuated or remodeled sex differences in malignant tissues compared with normal counterparts, with a large proportion of these loci involved in XCI-related regulation [36]. Similar sex-related divergence has been reported in glioblastoma, where methylation-driven activation in females is predominantly enriched in sex hormone–associated pathways [37]. Furthermore, additional evidence indicates that autosomal hypomethylation is a key determinant of poor survival in male patients with lung cancer [38]. Collectively, these observations support a contributory role for sex-biased DNA methylation in the differential engagement of cancer-relevant pathways and in disparities in tumor initiation and progression.

Mechanistically, these differences are driven in part by sex-specific regulation of DNA methyltransferases (DNMTs), including DNMT1, DNMT3A, and DNMT3B, whose expression and enzymatic activities have been reported to vary between sexes in multiple cancer types [39, 40]. An interesting study showed that pharmacological inhibition of DNMTs mimicked the effects of gonadal steroids, leading to masculinized neuronal markers and male sexual behavior in females [41]. In the context of cancer, studies have reported that DNMT3B expression is significantly higher in female liver tissue than in male liver tissue [40], and that DNMT3B levels are also markedly elevated in female compared with male patients with non-smoking–associated lung cancer [39]. This female-biased DNMT3B expression may drive distinct methylation landscapes that underpin the susceptibility differences observed in these malignancies.

#### Histone acetylation dynamics: hats and HDACs

Histone modifications—including acetylation, methylation, and phosphorylation—exhibit sex-specific patterns that regulate cancer progression.

Histone acetylation is a highly dynamic epigenetic modification that promotes chromatin accessibility and transcriptional activation, and its regulation exhibits pronounced sex dependency in cancer. Histone acetyltransferases (HATs), such as p300 and CBP, function as critical coactivators for estrogen and androgen receptors, enabling sex hormone–dependent transcriptional programs by acetylating histone tails at hormone-responsive enhancers and promoters [42]. In an oral squamous cell carcinoma mouse model, female mice exhibited higher levels of histone H3 acetylation (H3K9ac and H3K14ac) at the dysplasia stage compared with males. These hormone-influenced histone acetylation patterns have been considered an important factor contributing to the lower incidence of oral cancer in females [43]. In prostate cancer, histone H2A Lys130 acetylation is validated as an epigenetic mechanism promoting *de novo* androgen synthesis to overcome the pharmacological blockade of androgen biosynthesis [44].

While HATs facilitate gene activation, histone deacetylases (HDACs) counterbalance this process by enforcing gene repression. Several HDAC family members have been reported to display sex-biased expression or activity in tumors, contributing to differential regulation of cell cycle control, apoptosis, and immune-associated genes [45]. Importantly, HDAC-mediated regulation frequently intersects with androgen receptor signaling, particularly in male-predominant cancers such as prostate cancer, where AR–HDAC cooperation contributes to therapeutic resistance and to the development of drug-refractory tumors [46]. Recent evidence indicates that HDAC1-mediated remodeling of estrogen receptor α (ERα) expression contributes to the development of tamoxifen resistance in breast cancer [47]. These sex-dependent functions of HATs and HDACs not only shape transcriptional heterogeneity but also suggest that therapeutic responses to HDAC inhibitors may differ between male and female patients.

#### Histone methylation and sex chromosome dosage

Histone methylation provides an additional layer of complexity, as distinct marks can either activate or repress transcription depending on the genomic context. The histone methyltransferase EZH2, the catalytic component of the Polycomb repressive complex 2 (PRC2), mediates trimethylation of H3K27 and plays a central role in transcriptional silencing [48]. In male-biased cancers such as prostate cancer and melanoma tumor, EZH2 overexpression is frequently associated with aggressive disease and poor prognosis [49, 50].

In addition to methylation-dependent regulation, sex differences in histone demethylation represent another key epigenetic mechanism contributing to sexual dimorphism in cancer. The X-linked KDM6A (a histone demethylase) is expressed at higher levels in females, inhibiting tumor growth via H3K27me3 demethylation [51]. This gene has been identified as a sexually dimorphic gene, and studies have revealed that it plays a protective role against bladder cancer in females [52]. In males, the Y chromosome encodes histone demethylases such as KDM5D that are absent in females, and loss of KDM5D has been linked to poorer survival in male lung cancer patients [38].

Taken together, epigenetic writers and erasers establish sex-biased chromatin landscapes in cancer by integrating hormonal cues with sex chromosome–linked regulatory inputs. These mechanisms generate persistent transcriptional asymmetries that underlie sex-specific tumor initiation, progression, and treatment response across multiple cancer types.

### Epigenetic readers as integrators of sex signals

Epigenetic readers serve as critical molecular interpreters, translating established chromatin states into functional transcriptional outputs. Among these readers, bromodomain-containing proteins—particularly members of the Bromodomain and Extra-Terminal (BET) family such as BRD4—bind acetylated histones and promote transcriptional elongation at active regulatory regions. In osteosarcoma, androgen-induced upregulation of BRD4 facilitates malignant progression by engaging AR-dependent transcriptional programs, in part through the formation of an AR–BRD4 transcriptional complex that enhances PLCB4 expression [53]. In parallel, BRD3 has been shown to be selectively recruited to a subset of ERα binding sites enriched for active enhancer features, which cluster into putative super-enhancers and drive robust estrogen (E2)–responsive transcriptional programs, thereby promoting the progression of ER-positive breast cancer [54]. Beyond BRD3, BRD4 plays a central role in ER–dependent transcription by associating with acetylated chromatin at ERα-bound enhancers and promoting gene activation [55]. These observations raise the possibility that BET inhibitors may exhibit sex-biased efficacy or toxicity profiles, highlighting the need for sex-aware therapeutic evaluation.

### Sex-biased non-coding RNAs as epigenetic regulators in cancer

NcRNAs constitute a critical regulatory layer in epigenetic control by modulating chromatin structure, transcriptional activity, and the function of epigenetic machinery. Accumulating evidence indicates that both small ncRNAs, particularly microRNAs (miRNAs), and long non-coding RNAs (lncRNAs) exhibit pronounced sex-biased expression patterns in cancer, thereby contributing to sex-specific transcriptional programs and tumor phenotypes [56]. In a study of 111 cancer epigenetics studies, 63% investigated ncRNAs, with 28% reporting sex-specific ncRNA profiles [57]. These ncRNAs modulate epigenetic marks directly (e.g., lncRNAs interacting with chromatin modifiers) or indirectly (e.g., miRNAs targeting epigenetic regulators), contributing to sex disparities in cancer biology. These ncRNAs function not only as downstream effectors of sex hormone signaling, but also as upstream regulators that influence the activity, localization, and target specificity of epigenetic writers, erasers, and readers.

#### Sex-biased MicroRNAs and the miRNA-epigenetic axis

The miRNA landscape in cancer is fundamentally shaped by sex-linked genomic architecture; the X chromosome is significantly enriched with miRNAs compared to the Y chromosome, creating a baseline dosage disparity between sexes [58]. A liquid biopsy study identified multiple circulating miRNAs (e.g., miR-126-3p) that showed opposite expression changes in male versus female cancer patients compared to healthy controls, highlighting their potential as sex-aware diagnostic biomarkers [59]. Small RNA-sequencing identified 65 male-specific versus 12 female-specific deregulated miRNAs during progression to HCC, with many linked to ER signaling [60].

These sex-biased miRNAs can directly regulate epigenetic modifiers by targeting mRNAs encoding DNMTs, histone-modifying enzymes, or chromatin-associated proteins, thereby indirectly shaping genome-wide epigenetic landscapes [61]. For example, estrogen-responsive miRNAs have been shown to suppress the expression of DNMT and histone deacetylases, leading to sex-dependent differences in chromatin accessibility and gene expression [62]. In a mouse model, tobacco smoke exposure led to a female-specific suppression of tumor-suppressive miRNAs, which amplified tumor multiplicity in females via estrogen-receptor-targeted circuits [63]. Importantly, since estrogen signaling can epigenetically upregulate tumor-suppressive miRNAs in cancers such as HCC, combining hormone modulation with epigenetic inhibitors represents a promising, sex-aware therapeutic strategy [64].

#### Functional divergence of LncRNAs in sex-biased epigenetic landscapes

Long non-coding RNAs represent a particularly important class of sex-biased epigenetic regulators due to their strong association with sex chromosomes and hormone-responsive transcription. At the chromosomal level, lncRNAs impose fundamental constraints on chromatin dynamics. In females, the X-linked lncRNA *XIST* regulates XCI via DNA methylation and histone modifications [65], as discussed in detail in Sect. "X chromosome dysfunction". Beyond *XIST*, genetic variations (SNPs) within other lncRNA loci modulate colorectal neoplasia risk in a sex- and site-specific manner, with a protective effect observed mainly in females [66]. In contrast, male-biased lncRNAs contribute to distinct molecular signatures; for instance, an integrated analysis of head and neck cancer revealed six lncRNAs overexpressed in males that are associated with immunosuppression and metabolic rewiring [67]. Notably, the lncRNA *AL606489.1*, which is differentially expressed between sexes in lung cancer, and its high expression negatively correlates with survival exclusively in male patients [68]. These sex chromosome–linked lncRNAs exemplify how ncRNAs can impose sex-specific constraints on chromatin landscapes and transcriptional outputs.

Beyond chromosomal inheritance, autosomal lncRNAs integrate hormonal signals to amplify or remodel oncogenic epigenetic programs. A large repertoire of autosomal lncRNAs are regulated by estrogen or androgen signaling, reinforcing sex-biased transcriptional states through interactions with chromatin modifiers and enhancers [69]. Estrogen-induced lncRNAs in breast and gynecological cancers can influence histone modification patterns and enhancer activity, reinforcing female-biased transcriptional states [70]. In hormone-driven malignancies, these lncRNAs often function as “co-regulators” that stabilize hormone receptor activity. Androgen-responsive *PCA3* is highly enriched in male prostate tumors, where it modulates androgen receptor–dependent transcriptional programs by interacting with chromatin modifiers and transcriptional co-regulators [71]. Similarly, the lncRNA *Androgen Receptor Regulated Long Non-coding RNA 1* (*ARLNC1*) regulates androgen receptor signaling and is one of the most differentially expressed androgen receptor-regulated genes in prostate cancer. *ARLNC1* functions by binding to the androgen receptor, promoting its stability and enhancing transcriptional activity through a positive-feedback loop, thereby potentiating cell proliferation [72]. Similarly, in female-biased contexts, the lncRNA *Homeobox transcript antisense intergenic RNA* (*HOTAIR*) acts as a central node bridging estrogen signaling and metastatic progression. *HOTAIR* not only upregulates nuclear ER levels but also interacts with chromatin to facilitate metastasis via the remodeling of local epigenetic states [73, 74]. Estrogen stimulates *HOTAIR* via the G protein-coupled receptor and suppresses miRNA miR-148a, which negatively affects *HOTAIR* levels [75].

Crucially, the effects of sex hormones and their interacting lncRNAs can also be sexually dimorphic on non-reproductive cancers. Estrogen-receptor-associated lncRNAs can drive progression in traditionally “non-hormonal” tissues. ERβ-induced MALAT1 suppresses miR-145-5p leading to increased expression of the oncogene NEDD9 and thereby promoting the progression of NSCLC in female [76]. Conversely, androgen-associated lncRNAs contribute to the male-biased aggressiveness of several cancers. In melanoma, the lncRNA *SRA-like Non-coding RNA* (*SLNCR*) binds directly to the AR to activate invasion-related transcription, leading to a poorer prognosis for men [77]. Interestingly, some lncRNAs function as protective “epigenetic brakes” whose loss contributes to sex disparities. the lncRNA *Suppressing Androgen Receptor in Renal Cell Carcinoma* (*SARCC*) suppresses renal cancer growth by inhibiting AR functions, specifically through stabilizing the AR protein in males, leading to a repression of miR-142-3p. When overexpressed, SARCC inhibits downstream signals in the AKT, MMP, K-RAS, and P-ERK pathways; however, its downregulation in males releases this suppression, potentially explaining the significantly higher incidence and aggressiveness of renal cancer in men compared to women [78].

Collectively, sex-specific regulation by epigenetic writers, erasers, and readers establishes durable transcriptional asymmetries that shape cancer risk, progression, and treatment response. Because epigenetic modifications are inherently reversible, these mechanisms provide a compelling rationale for incorporating biological sex into epigenetic biomarker development and therapeutic strategies, a concept that will be further explored in the context of precision oncology. A 2021 Phase II clinical trial in relapsed/refractory Hodgkin lymphoma investigated the combination of a PD-1 inhibitor (camrelizumab) with the DNMT inhibitor decitabine. In the arm receiving the PD-1 inhibitor alone, female patients had a significantly longer progression-free survival (PFS) than male patients. Remarkably, the addition of decitabine eliminated this sex disparity, with both sexes deriving a strong PFS benefit from the combination therapy. This suggests that DNMT inhibition may overcome a relative resistance to immunotherapy in male patients [79].

## Sex hormone-mediated mechanisms of cancer sex differences

### Estrogen and ER

Differences in circulating sex hormone profiles are a key driver of cancer-related sex disparities, with significant variations in the type, concentration, and biological actions of sex hormones between males and females. Estrogen—predominantly a female sex hormone—plays an indispensable role in sexual and reproductive development, with four major natural isoforms (estrone [E1], estradiol [E2], estriol [E3], and estetrol [E4]) exhibiting temporally and spatially regulated expression patterns.

E2 is the most biologically active estrogen: primarily produced by the ovaries in premenopausal women (with minor synthesis in the adrenal gland, testes, and adipose tissue), it regulates the development of female secondary sexual characteristics. Postmenopausally, ovarian E2 production declines, and small amounts are synthesized in extragonadal tissues (e.g., brain, bone, adipose tissue) [80–83]. E1, a weaker estrogen with lower affinity for ERs than E2, is produced by the ovaries and via extragonadal conversion of androstenedione—this conversion sustains E1 levels in postmenopausal women, making it the dominant circulating estrogen in this group [80]. E3 and E4 are pregnancy-specific estrogens: E3 is synthesized by the placenta (undetectable in non-pregnant women) and acts as a short-acting ER agonist-antagonist; E4 is produced by the fetal liver, shares structural similarities with E3, and is only detectable during gestation [81]. Notably, males also produce low levels of estrogen (via adrenal androgen aromatization and testicular synthesis), with concentrations approximately 10–20% of those in premenopausal women—this underappreciated pool of male estrogen contributes to sex-specific tumor regulation (e.g., ERβ-mediated tumor suppression in male lung cancer) [80–83].

Notably, estrogen exerts its biological effects through binding to ERs, which fall into two functional categories. (1) Nuclear-initiated steroid signaling (NISS) is mediated by classical nuclear ERs (ERα and ERβ), which form homodimers or heterodimers upon estrogen binding. This complex binds to DNA estrogen response elements (EREs) or interacts with transcription factors (e.g., AP-1, SP-1) to regulate gene transcription, thereby exerting genomic effects [82, 84, 85]. (2) Membrane-initiated steroid signaling (MISS) is mediated by membrane-localized ERs, including membrane isoforms of nuclear ERs and GPR30/GPER1 (a G protein-coupled receptor). Estrogen binding activates rapid non-genomic signaling via kinases such as PI3K and MAPK, which regulates cell signaling, secretion, and ion channel activity [84, 85].

ERα and ERβ display tissue-specific expression patterns and exert opposing roles in tumorigenesis. ERα is highly expressed in reproductive organs (e.g., breast, endometrium) and primarily exerts pro-tumorigenic effects in most cancers, though it confers tumor-suppressive functions in the esophagus, stomach, and liver [86]. ERβ is enriched in the male reproductive system, central nervous system (CNS), lung, and immune system; its role is cancer-type-specific—serving as a tumor suppressor in ovarian cancer, prostate cancer, and cutaneous melanoma, yet a tumor promoter in endometrial cancer and bladder cancer—with anti-proliferative activities predominating in most contexts [86].

### Androgen and androgen receptors

Androgens are the dominant sex hormones in males, while present at lower levels in females, and consist of four major isoforms—testosterone (T), dihydrotestosterone (DHT), androstenedione, and dehydroepiandrosterone (DHEA). 90% of androgens in males are produced by the testes, with minor contributions from the adrenal cortex, whereas females synthesize androgens exclusively through peripheral DHEA metabolism [81, 87–89].

Testosterone is the most abundant androgen in males, maintaining secondary sexual characteristics and having the highest serum concentration in adult males. It is converted to dihydrotestosterone (DHT)—the most potent androgen, despite serum concentrations approximately 10% of those of testosterone—via 5α-reductase [87]. Androstenedione and dehydroepiandrosterone (DHEA) possess weak androgenic activity (10% and 5% of that of testosterone, respectively)—androstenedione interconverts with testosterone and is aromatized to estrogen, whereas DHEA acts as a precursor for both androgens and estrogens [81, 89]. Critically, DHT cannot be aromatized to estrogen, thereby functioning as a male-specific driver of androgen-dependent cancers (e.g., prostate cancer) [81, 87–89].

The AR—primarily cytoplasmic—translocates to the nucleus upon androgen binding, where it interacts with androgen response elements (AREs) to regulate gene expression. Like ERs, the AR exerts dual functional modes. (1) Classical nuclear signaling: The AR acts as a transcription factor to activate oncogenes (e.g., MYC, BCL-2, KLK3/PSA) in prostate cancer, thereby driving malignant proliferation [81, 90]. (2) Non-classical membrane signaling: Membrane-localized AR binds to G protein-coupled receptors to trigger rapid signaling cascades (e.g., protein synthesis, kinase activation), which enhances tumor invasion [90]. Androgens also modulate immune function: in contrast to estrogen’s immune-promoting effects, they exert immunosuppressive actions by regulating the differentiation of AR-positive immune cells and the secretion of anti-inflammatory cytokines [89].

In tumor cells, the role of androgens varies by tumor type and hormone concentration. In PCa, androgens act as carcinogens regardless of concentration, via the canonical “androgen-AR-ARE” axis. In castration-resistant PCa (CRPC), aberrant AR activation (e.g., AR amplification, T878A mutation, AR-V7 splice variant) sustains oncogenic signaling under low androgen conditions, driving treatment resistance [81]. AR-V7—lacking the ligand-binding domain and exhibiting constitutive activity—is detected in approximately 30% of CRPC patients and confers resistance to second-generation anti-androgens.

Mapping hormone receptor expression and downstream signaling pathways in both hormone-dependent and hormone-independent cancers can enable sex-specific endocrine interventions and combinatorial regimens, enhancing efficacy while minimizing hormone-driven adverse effects.

## Sex differences directly regulate tumor cell function: a multidimensional perspective

Sex-specific disparities in tumor cell functions—including proliferation, metabolism, and migration—are not isolated phenomena; instead, they are orchestrated by the combined regulatory actions of sex hormones and sex chromosome genes. Building upon these foundational regulatory mechanisms, this chapter aims to dissect the multidimensional pathways through which sex differences modulate tumor cell behavior, with a focus on integrating the synergistic effects of hormonal and genetic factors.

**Fig. 1:**
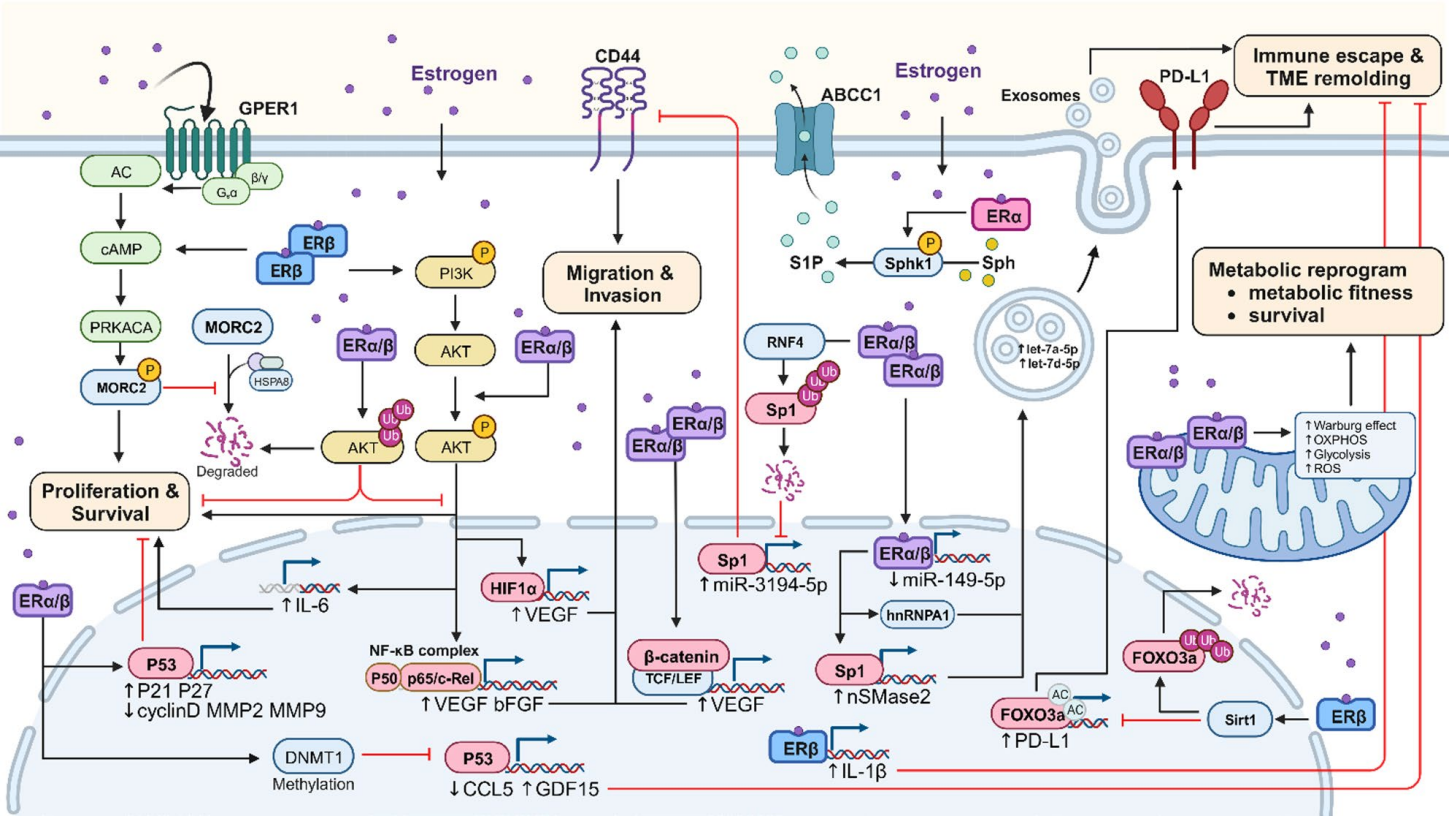
Estrogen on pathways of cancer cells

Notably, sex hormones exert direct regulatory effects on tumor cell metabolism and functional phenotypes—key contributors to cancer-related sex disparities. Upon binding to hormone receptors (HRs), sex hormones (primarily estrogen) modulate tumor cell functions through multi-layered regulation: they alter the expression of tumor-associated genes, regulate the abundance and activity of key proteins/RNAs, and reprogram critical signaling pathways through both genetic (e.g., transcriptional regulation) and non-genetic (e.g., post-translational modification) mechanisms. Collectively, these regulatory effects underpin the sex-specific functional phenotypes of tumor cells documented in both preclinical models and clinical observations (Fig. 1).

Estrogen affects gene expression and pathways in cancer cells, leading to both pro- and anti-tumoral effects. Binding to estrogen receptors (ER), it induces anti-tumor effects via the p53/p21 and p27 pathway, inhibiting the expression of cyclin D1 and MMP. Additionally, it can induce the release of IL-1β in tumors, which recruits anti-tumor neutrophils to enhance innate immunity and inhibit metastasis. Estrogen cypionate (ECP) contributes to this effect by increasing the ubiquitination level of AKT in cancer cells, subsequent inhibition of the PI3K/AKT/mTOR pathway. On the other hand, the promotion of tumor progression by estrogen involves complex signaling pathways. When estrogen binds to GPER1, it phosphorylates MORC2 by activating PRKACA, which protects MORC2 from lysosomal degradation. MORC2 upregulation promotes cancer cell proliferation and drug resistance. Estrogen stimulates IL6 expression through ERβ via MAPK/ERK and PI3K/AKT pathways, contributing to increased growth and proliferation. Furthermore, estrogen induces the degradation of Sp1, leading to decreased miR-3194-5p and increased CD44 expression, which facilitates cancer migration. Estrogen’s influence extends to angiogenesis and metastasis through pathways promoting VEGF secretion, involving the NF-κB、β-catenin and PI3K/HIF-1. Estrogen enhances tumor metabolism by increasing ER expression, stimulating oxidative phosphorylation (OxPhos), and promoting glycolytic metabolism. It can also alter mitochondrial function, increase oxidative stress, and affect invasion and motility. Estrogen regulates the secretion of cytokines. E2 reduces p53 expression, resulting in increased CCL5 and decreased GDF15, promoting the polarization of M2 macrophages, which can lead to a poor prognosis. Estrogen affects immune escape, TME remodeling through the SIRT1 axis, leading to the degradation of FOXO3a, increased PD-L1 expression, subsequent immune escape and metastasis, and extracellular vesicle (EV) secretion. It also promotes drug resistance, particularly through enhanced sphingosine-1-phosphate (S1P) export.

### Inhibiting tumor development

Sex hormones can inhibit tumor initiation and progression, exerting anti-tumor effects. Among these, 17β-estradiol (E2) prominently exerts anti-tumor effects across multiple cancer types. Studies have demonstrated that E2 significantly suppresses the proliferation of LoVo colorectal cancer cells by increasing the protein levels of p53, p21, and p27, which subsequently downregulates cyclin D1 expression. Additionally, E2 reduces the expression of matrix metalloproteinases (MMPs) and plasminogen activator (PA) systems via p53 pathway activation—this inhibits tumor cell motility and impedes tumor progression and metastasis [91]. Beyond endogenous estradiol, estradiol cypionate (ECP)—a synthetic ester of estradiol (estradiol 17-β-cyclopentylpropionate)—exerts anti-tumor effects in gastric cancer. ECP enhances AKT ubiquitination levels in gastric cancer cells, triggering AKT degradation via the proteasome pathway. Reduced AKT protein levels inhibit overactivation of the PI3K-AKT-mTOR signaling pathway, thereby suppressing gastric cancer cell proliferation and promoting apoptosis [92]. Notably, activation of ERs in tumors induces the release of interleukin-1β (IL-1β). Elevated IL-1β concentrations in the TME recruit anti-tumor neutrophils, which enhance innate immunity and suppress the metastasis and lung colonization of triple-negative breast cancer (TNBC) and melanoma cells [93].

### Promotion of tumor cell proliferation and migration

Sex differences in tumor cell proliferation and migration are tightly linked to the context-dependent actions of sex hormones—with 17β-estradiol (E2) and androgens (via AR) exerting distinct, cancer-type-specific regulatory effects. For most hormone-independent cancers (e.g., gastric cancer, colorectal cancer), women exhibit lower incidence and better prognosis than men; however, in certain cancers (e.g., NSCLC, hormone-dependent cancers such as breast cancer), E2-driven promotion of tumor cell proliferation and migration contributes to worse clinical outcomes in women.

E2 mediates pro-tumorigenic effects on proliferation through multiple receptor-dependent mechanisms. Upon binding to the G protein-coupled estrogen receptor 1 (GPER1), E2 activates PRKACA—a subunit of protein kinase A (PKA)—which phosphorylates MORC2 (microrchidia family CW-type zinc finger 2) at the T582 residue. This phosphorylation shields MORC2 from chaperone-mediated autophagy (CMA)-dependent lysosomal degradation; elevated MORC2 levels then promote breast cancer cell proliferation and enhance resistance to endocrine therapies, driving tumor progression [94]. In NSCLC, E2 acts via ERs—specifically ERβ—to stimulate interleukin-6 (IL-6) expression through the MAPK/ERK and PI3K/AKT signaling pathways; IL-6 then further activates downstream pro-proliferative cascades, supporting the growth and expansion of NSCLC cells [95].

E2 also facilitates cancer cell migration and invasion across diverse tumor types by reprogramming key molecular pathways. In lung cancer, E2 downregulates the expression of microRNA-3194-5p (miR-3194-5p)—a microRNA that normally targets the transcription factor Sp1 for degradation; decreased miR-3194-5p levels lead to Sp1 accumulation, which in turn upregulates CD44 (a cancer stem cell marker associated with migration), ultimately enhancing lung cancer cell motility [96]. In endometrial carcinoma, E2 activates the serine/threonine kinase Akt, which in turn triggers NF-κB pathway activation; this upregulates the secretion of vascular endothelial growth factor (VEGF) and basic fibroblast growth factor (bFGF), inducing angiogenesis and promoting tumor growth, invasion, and migration [97]. Even in prostate cancer, E2 contributes to aggressive behavior: after binding to ERs (primarily ERα), E2 upregulates the expression of β-catenin and VEGFα—two molecules critical for cell adhesion and angiogenesis—thereby enhancing prostate cancer cell invasion and migration [98]. In breast cancer, E2 further drives metastasis by upregulating hypoxia-inducible factor-1 (HIF-1) via the PI3K pathway; HIF-1 promotes VEGF secretion, accelerating angiogenesis and supporting distant tumor colonization [99].

### Metabolic reprogramming of tumor cells

Sex hormones drive tumor progression by orchestrating metabolic reprogramming—a hallmark of cancer—with 17β-estradiol (E2) and androgens (via AR) imposing distinct, sex- and cancer-type-specific metabolic profiles. Notably, this reprogramming is not solely mediated by sex hormones; sex chromosome genes further contribute to sex disparities in tumor metabolism, highlighting a synergistic interplay between hormonal and genetic regulators.

E2 modulates tumor metabolism primarily through ER-dependent regulation of energy pathways, with effects varying by cancer type. In ER^+^ MCF7 breast cancer cells, E2 upregulates ER expression, which in turn co-stimulates both oxidative phosphorylation (OxPhos) and glycolytic metabolism. This dual activation of energy pathways provides sufficient ATP and biosynthetic intermediates to support tumor growth, invasion, and metastasis [100]. In obese breast cancer patients, ELIT—a combination therapy containing 17β-estradiol—exerts context-dependent metabolic effects: in breast cancer cell lines with a high ERα/ERβ ratio, ELIT reduces mitochondrial function, increases oxidative stress, and enhances cell invasion/motility; in contrast, cell lines with a low ERα/ERβ ratio show no such metabolic or functional changes—underscoring the critical role of ER subtype balance in E2-mediated metabolic regulation [101]. Beyond breast cancer, E2 drives metabolic reprogramming in cervical cancer by promoting the Warburg effect (aerobic glycolysis despite adequate oxygen): it upregulates the expression of mitochondrial respiratory chain complexes and key glycolytic enzymes, simultaneously boosting mitochondrial respiration efficiency and glycolytic flux. This dual metabolic adaptation protects cervical cancer cells from stress-induced death during malignant transformation, facilitating aggressive progression [102].

### Promotion of tumor cell cytokine secretion

Estrogen can promote the malignant progression of tumors by regulating the secretion of tumor cytokines. In breast cancer cells, E2 upregulates TNFα expression and secretion after binding with ERα. TNFα then stimulates the surrounding ester-producing adipose fibroblasts to produce estrogen. Positive feedback occurs between E2 and TNFα, which maintains high concentrations in the ER^+^ breast tumor microenvironment and promotes poor prognosis [103]. In addition, E2 reduces the expression of p53 by upregulating the level of DNA methyltransferase 1 (DNMT1), leading to an increase in CCL5 and a decrease in GDF15, promoting the polarization of M2 macrophages and resulting in a poor prognosis in female lung cancer patients [104]. After the same stimulation by estrogen, E2 induced the upregulation of p53 expression in colorectal cells and played a tumor suppressor role. However, E2 induces a decrease in p53 expression in lung cancer cells and promotes tumor progression [91]. This difference in cellular estrogen action may be related to cell type and cellular ER expression.

### Promotion of tumor cell immune escape and TME remodeling

Sex hormones modulate tumor progression not only by directly regulating tumor cell functions but also by reshaping the TME and facilitating immune escape—with 17β-estradiol (E2) and androgens (via AR) driving sex-specific immunosuppressive landscapes. E2 promotes immune escape and TME remodeling through receptor-dependent molecular cascades across multiple cancers. In NSCLC, E2 upregulates ERβ, which in turn activates the sirtuin 1 (SIRT1) signaling axis. SIRT1 decreases the acetylation level and enhances the ubiquitination level of forkhead box O3a (FOXO3a), triggering FOXO3a degradation. With FOXO3a—a transcriptional repressor of PD-L1—depleted, PD-L1 expression on NSCLC cells is significantly upregulated; this inhibits the cytotoxic activity of CD8^+^ T cells, enabling tumor immune escape and promoting distant metastasis [105].

In breast cancer, E2 modulates TME composition by regulating extracellular vesicle (EV) secretion: upon binding to ERs, E2 downregulates miR-149-5p, which relieves translational repression of the transcription factor SP1. Elevated SP1 then upregulates neutral sphingomyelinase 2 (nSMase2)—an enzyme critical for EV biogenesis. Concurrently, reduced miR-149-5p promotes the expression of heterogeneous nuclear ribonucleoprotein A1 (hnRNA1), which facilitates the loading of let-7 family miRNAs into EVs. Breast cancer cells release these let-7-enriched EVs to polarize tumor-associated macrophages (TAMs) toward an M2-like pro-tumorigenic phenotype, thereby remodeling the TME to support tumor growth [106].

### Promotion of carcinogenesis and tumor cell drug resistance

When estrogen metabolism is imbalanced, higher levels of 4-OHE1 (E2), E1 (E2)−3, and 4-Q are produced. Binding of E1(E2)−3,4-Q to DNA can result in errors at this binding site during subsequent repair, causing gene mutation and carcinogenesis [107]. Moreover, the effect of E2 on tumors also manifests as the promotion of tumor drug resistance. After stimulating ERα, E2 enhances the export of sphingosine 1-phosphate (S1P) in MCF-7 cells through ABCC1 and ABCG1 and promotes the progression and drug resistance of breast cancer [108].

The effect of hormones on tumors is affected by tumor type, hormone concentration, receptor distribution, and other factors and manifests as pro-tumor and anti-tumor effects. These tumors can be divided into hormone-dependent tumors and hormone-independent tumors. The biological behavior of breast cancer, endometrial cancer and prostate cancer, which are directly regulated by ER, are hormone-dependent tumors that directly enhance malignant behavior after being stimulated by hormones. However, other types of ER-independent malignant tumor cells are also affected by hormones and undergo cancer progression or suppression in response to the action of hormones, depending on the cell type. Although epidemiological data show that estrogen mainly plays a protective role, most of the current research findings still suggest that elevated estrogen levels can promote tumor progression and aggressiveness by affecting tumor-related genes to regulate proliferation, migration, drug resistance, and other biological behaviors; on the other hand, high estrogen levels can also affect the tumor microenvironment through the production of cytokines or vesicles by tumor cells, promoting tumor progression. Such differential distribution of estrogen between the sexes and the unique effects of estrogen on tumors contribute to sex differences in cancer incidence between men and women.

## Sex differences affect tumor development via immune system imbalances

Sex differences in the human immune system and immune responses—key mediators of tumor development—are regulated not only by sex hormones but also by sex chromosome genes and gut microbiota, ultimately driving divergent immune profiles between males and females. In general, females exhibit more robust immune responses than males—a pattern reflected in the female predominance of most autoimmune diseases. This sex bias arises from complex interactions between genes, hormones, and the environment, with sex hormones contributing substantially to this variance.

Furthermore, sex chromosome genes play a crucial role in shaping immune responses. For instance, genes located on the X chromosome, such as those involved in immune regulation and antibody production, can influence the strength and efficacy of the immune response in females. Genetic abnormalities, including X chromosome aneuploidies or deletions, can lead to dysregulation of these immune-related genes, further complicating the immune landscape. This can result in altered immune functionality and increased susceptibility to autoimmune conditions, which are more prevalent in females.

**Fig. 2:**
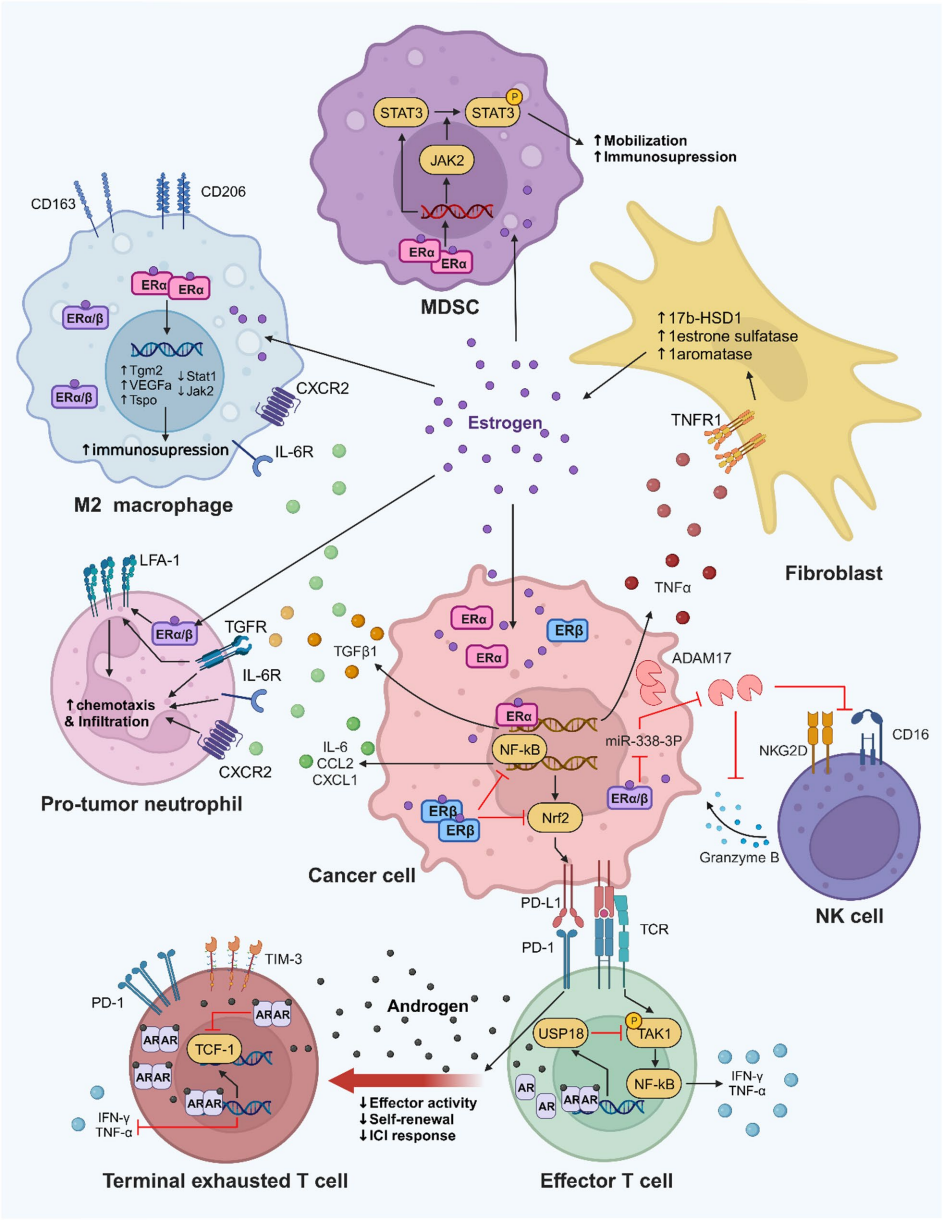
The Influence of Sex Hormones on Cell Subpopulations in the Tumor Microenvironment

Sex hormones drive these immune differences by modulating the development, differentiation, polarization, and function of innate and adaptive immune cells—including regulating phagocytic activity, cytokine secretion, and antibody production. Specifically, 17β-estradiol (E2) enhances both humoral and cellular immune responses, whereas androgens typically inhibit immune cell activity and suppress immune responses. These opposing hormonal effects are a primary driver of sex-based immune disparities, which in turn shape tumor progression by altering anti-tumor immunity and the TME [53, 54]. (Fig. 2)

Estrogen and androgens have complex effects on cells within the tumor microenvironment, affecting various cellular processes. By binding to ERβ, estrogen inhibits NF-κB activity in cancer cells, subsequently reducing PD-L1 expression and secretion of IL-6, CCL2, and CXCL1. This suppresses the immunosuppressive features of the tumor microenvironment (TME), including neutrophil infiltration, M2 polarization of macrophages, and myeloid-derived suppressor cells (MDSCs), leading to the activation of cytotoxic antitumor immunity and inhibition of tumor progression.

On the other hand, estrogen has been found to promote tumors through multiple pathways. When it binds to ER, it biases macrophages towards an immunosuppressive state and enhances pSTAT3 activity in myeloid progenitor cells. This promotes MDSC infiltration into the tumor site, which facilitates the establishment of an immunosuppressive TME. Furthermore, estrogen increases the secretion of TGFβ1, leading to an increased accumulation of LFA-1 + neutrophils. Estrogen down-regulates miR-338-3p in cancer cells, resulting in increased expression of ADAM17, which impairs natural killer (NK) cell activity. Additionally, estrogen-driven mechanisms involve upregulating TNFα in cancer cells, creating a positive feedback loop with surrounding fibroblasts and sustaining high estrogen concentrations in the tumor microenvironment.

Androgens, on the other hand, inhibit anti-tumor immune responses. Androgen receptors (AR) activate ubiquitin-specific protease 18 (USP18), which inhibits the phosphorylation of transforming growth factor kinase 1 (TAK1) and activates the NF-κB signaling pathway in anti-tumor T cells. Additionally, androgens contribute to CD8^+^ T cell exhaustion by directly regulating Tcf7 which involved in determining the early fate of activated CD8^+^ T cells, and inhibiting the expression of IFNγ in CD8^+^ T cells.

### Anti-tumor immune response

Sex differences significantly impact immune responses and cancer outcomes, with emerging evidence highlighting the influence of genetic and epigenetic factors. Notably, the LOY in PBMCs is the most prevalent somatic alteration in men and is associated with higher mortality from epithelial cancers. Comprehensive analyses reveal that LOY is prevalent in both malignant epithelial and immune cells, with LOY in these cells correlating with a shift towards immunosuppression in CD4^+^ and CD8^+^ T cells, thus contributing to poorer survival outcomes [109].

In glioblastoma (GBM), distinct immunological sex differences are observed, with male mice exhibiting accelerated tumor growth and increased T cell exhaustion, partly regulated by genes escaping X chromosome inactivation, such as *Kdm6a* [110]. Furthermore, while natural killer (NK) cells are numerically increased in males, their effector function is diminished, indicating a complex interplay between sex, immune cell functionality, and cancer progression [111]. Together, these findings illustrate how genetic anomalies and sex differences in immune cell behavior can shape tumor immunity, contributing to the variance in cancer incidence and outcomes between genders.

Many studies have shown that sex hormones not only play a role in the normal immune system but also have an impact on tumor immunity in many different ways. Sex hormones can play a protective role in the body by affecting the innate or adaptive immune system against tumors. Estrogen can inhibit NF-κB activity in STAT3-deficient *K-Ras* lung adenocarcinoma cells; suppress the immunosuppressive properties of the TME by suppressing the infiltration of neutrophils, reducing the M2 polarization of macrophages, and inhibiting myeloid-derived suppressor cells (MDSCs); and ultimately activate cytotoxic anti-tumor immunity to inhibit malignant tumor progression [112].

In a mouse model of MC38 colon cancer, combination treatment with E2 and an anti-PD-L1 antibody reduced PD-L1 expression, suppressed the immunosuppressive macrophage population (CD11b^+^F4/80^+^) and cancer-associated fibroblasts (CAFs), and increased the population of M1 TAMs (CD11b^+^F4/80^+^CD86^+^), which significantly inhibited tumor growth [113].

Sex hormones can affect innate and adaptive immunity by regulating immune cells and the immune microenvironment to exert anti-tumor effects. However, most of the current research on the effect of sex hormones on the occurrence and development of cancer has focused mainly on the role of sex hormones in promoting tumors through tumor immunity. We explore this phenomenon in more detail below.

### Pro-tumor immune response

In addition to exerting anti-tumor protective effects, sex hormones can facilitate cancer progression by suppressing anti-tumor immune responses and expanding immunosuppressive cell populations.

After binding to ERα, E2 induces an immunosuppressive state in macrophages, promotes the establishment of an immunosuppressive TME, and leads to CD8^+^ T-cell dysfunction and immune checkpoint blockade (ICB) resistance, which promotes melanoma growth [114]. E2 could also downregulate the level of the tumor suppressor miR-338-3p and increase the expression of disintegrin and metalloprotease-17 (ADAM17) in breast cancer cells. The subsequent downregulation of NKG2D, Granzyme B, and CD16 in NK cells can impair the activity of NK cells, promoting high activity, proliferation, and development of breast cancer cells [115].

Through increased expression of Janus kinase 2 (JAK2) and upregulation of total STAT3, estrogen enhances pSTAT3 activity in myeloid progenitor cells, promotes MDSCs to the tumor site, and induces an immunosuppressive TME, accelerating the progression of hormone-independent types of tumors [116]. It is interesting to note that estrogen receptors α promote M2 macrophage polarization in melanoma by inhibiting STAT1 and JAK2 transcription [114]. These results suggest that the effects of E2 and ER on STAT and JAK signaling pathways may be multifaceted. E2 also enhanced tumor invasion and metastasis through neutrophils. E2 increased TGFβ1 secretion in tumor and promoted neutrophil infiltration in the microspheres of breast tumors, leading to a high degree of metastasis of noninvasive cancer cells [117].

In addition to estrogen acting on tumor immunity, androgens also contribute to tumor progression by influencing immune responses. The androgen-activated androgen receptor upregulates the expression of ubiquitin-specific protease 18 (USP18), thereby inhibiting the phosphorylation of transforming growth factor kinase 1 (TAK1) and activation of the *NF-κB* signaling pathway in antitumor T cells, inhibiting the anti-tumoral effect of T cells and promoting tumor progression [118].

Androgens also promote CD8^+^ T-cell depletion by binding to AR through direct regulation of Tcf7, which is involved in determining the early fate of activated CD8^+^ T cells and inhibiting the expression of IFNγ in CD8^+^ T cells, promoting CD8^+^ T-cell exhaustion and reducing the sensitivity to immune checkpoint blockade therapy [119, 120]. However, the literature presents opposing findings regarding the mode of Tcf7 regulation. Although the conclusion is that AR inhibits the anti-tumor function of CD8^+^ T cells, Deng et al. found that AR signal regulates the differentiation of stem-like CD8^+^ T cells into to terminally exhausted CD8^+^ T cells by inhibiting Tcf7 transcription [121]. These discrepancies may arise from differences in tumor type–specific microenvironments (including cytokine profiles) and variation in AR co-regulator expression patterns within CD8^+^ T-cell subsets of different lineages. Understanding these context-dependent mechanisms is essential for accurately interpreting AR’s role in tumor immunity and may have implications for designing combination therapies that target androgen signaling in diverse tumor types.

Although estrogen plays a positive role in activating the immune system under physiological conditions, it mainly exerts a negative tumor-promoting function on tumors. Most estrogen exerts its tumor-promoting effect by affecting the function of myeloid cells, especially macrophages, while androgen affects the adaptive immune response, especially the killing function of CD8^+^ T cells, which play important roles in inhibiting immune function and promoting tumors. In addition, some studies have tried to use E2 combined with an anti-PD-L1 antibody to treat tumors in animals, which provides a theoretical basis for later clinical trials. In fact, the finding that androgen induces CD8^+^ T-cell exhaustion suggests that the use of androgen antagonists combined with immunotherapy may also be a promising direction for exploring cancer therapy.

It is interesting to note that estrogen receptors α promote M2 macrophage polarization in melanoma by inhibiting STAT1 and JAK2 transcription [114]. However, in another study, estrogen receptor α enhanced the immunosuppressive function of MDSC by enhancing JAK2 and SRC activity to activate the STAT3 pathway in human and mouse myeloid progenitor cells [116].

## Sex differences affect tumor development via tumor stromal cells

Functional differences in tumor stromal cells are key targets of sex-related regulatory factors (e.g., sex hormones and chromosomal genes). This section focuses on their regulatory roles in tumor progression, dissecting how sex-biased modulation of stromal cell function drives disparities in tumor initiation, invasion, and metastasis. Tumor stromal cells primarily comprise cancer-associated fibroblasts (CAFs), vascular endothelial cells, mesenchymal stromal cells (MSCs), and immune cells; they play critical roles in promoting tumor angiogenesis, sustaining tumor growth, and facilitating invasion. Sex-related factors—including sex hormones and non-hormonal regulators (e.g., sex chromosome genes, gut microbiota metabolites)—shape tumor development by modulating the biological behavior of these stromal components [122].

CAFs are a pivotal subset of the TME, with functions spanning extracellular matrix (ECM) remodeling, angiogenesis regulation, paracrine crosstalk with cancer cells, and immune cell recruitment via cytokine secretion [123]. CAF gene expression profiles and functional phenotypes vary by tumor type and sex, driven by both hormonal and genetic factors. 17β-estradiol (E2)—a major female sex hormone—contributes to cancer sex disparities by targeting CAFs: E2 stimulates ERα-positive CAFs to upregulate CD147 expression, which in turn enhances the expression levels of matrix metalloproteinases (MMP2 and MMP9) in both CAFs and gastric cancer cells, ultimately promoting gastric cancer cell invasion and migration [124]. Additionally, E2 induces CAFs to secrete interleukin-6 (IL-6), which activates the STAT3 pathway in gastric cancer cells and accelerates their proliferation and invasion [125]. In breast cancer, E2 further regulates CAF function by modulating miRNA expression, controlling CAF proliferation, motility, and pro-angiogenic activity to drive tumor progression and metastasis [126].

Tumor angiogenesis—critical for supplying oxygen and nutrients to cancer cells and enabling metastatic dissemination—is a key process influenced by sex differences, involving both hormonal and gut microbiota-dependent mechanisms. Higher microvessel density in tumor tissue is consistently associated with poor patient prognosis across cancer types [127]. E2 drives angiogenesis by targeting vascular endothelial cells: it upregulates aquaporin-1 (AQP1) expression in human umbilical vein endothelial cells (HUVECs), thereby enhancing endothelial cell migration and tube formation. This effect promotes angiogenesis in breast and endometrial cancer, supporting tumor growth, invasion, and metastasis [128].

Collectively, tumor stromal cells are indispensable for cancer development, with their function shaped by the combined effects of sex hormones, X chromosome genes, and gut microbiota metabolites. 17β-estradiol (E2) regulates CAF and vascular endothelial cell behavior via ER-mediated gene expression, cell secretion, and signal transduction, indirectly promoting tumor progression—with the E2-ER-STAT3 axis operating in both tumor cells and CAFs, highlighting STAT3 as a key node in stromal-tumor crosstalk. The inclusion of non-hormonal regulators (e.g., X-linked FOXP3, gut-derived TMAO) further underscores the complexity of sex-related stromal modulation. Targeting these sex-biased stromal pathways—for example, developing antagonists against the E2-ER-STAT3 axis or inhibiting TMAO production—may provide more precise, sex-tailored strategies for tumor intervention.

## Sex differences in response to cancer treatment

Sex differences influence not only the initiation and progression of various cancers but also the efficacy of tumor therapies. These treatment disparities arise from three interconnected factors: the divergent effects of sex hormones and their receptors, sex-biased immune responses, and physiological differences that alter drug pharmacokinetics and pharmacodynamics. For instance, in ICB therapy for melanoma and NSCLC, men exhibit better outcomes than women [129]; conversely, standard glioblastoma treatment (e.g., temozolomide-based therapy) is more effective in women than in men [130].

Sex hormones are a primary driver of treatment efficacy disparities, acting through direct effects on tumor cells, modulation of anti-tumor immunity, and regulation of drug metabolism. In urothelial cancer (UC) patients receiving ICB, high estrogen levels correlate with poor survival [131]. In ER-positive breast cancer, suppressing estrogen levels (e.g., via aromatase inhibitors) enhances sensitivity to ionizing radiation [132]. In melanoma mouse models, 17β-estradiol (E2) binds to ERα to polarize macrophages toward an immunosuppressive phenotype, promoting CD8^+^ T-cell exhaustion and ICB resistance—partially explaining why men derive greater benefit from ICB [114]. Beyond immunity, E2 regulates drug metabolism by altering hepatic CYP enzyme expression via ER signaling, accelerating the breakdown of anticancer drugs. It also modulates downstream miRNA levels to regulate genes involved in cell proliferation, differentiation, and apoptosis, fostering resistance to chemotherapeutics (e.g., cyclophosphamide, methotrexate, paclitaxel) [133].

Notably, physiological sex differences—many of which are regulated by sex hormones—further shape treatment responses by disrupting pharmacokinetic and pharmacodynamic processes.

### Fat distribution: hormone-regulated drug sequestration and metabolism

Sex hormones orchestrate distinct patterns of fat distribution, which in turn modulate the pharmacokinetic behavior of lipophilic anticancer drugs. Estrogen promotes subcutaneous fat accumulation, resulting in an approximately 10% higher average total body fat content in women compared to men [131]. Critically, this expanded subcutaneous fat compartment acts as a physiological reservoir for lipophilic chemotherapeutic agents—such as paclitaxel. Consequently, paclitaxel demonstrates a prolonged pharmacokinetic half-life in women, which not only elevates the risk of cumulative toxicity but also necessitates individualized dose adjustments to balance efficacy and safety. In contrast, androgens favor visceral fat deposition in men, a distribution pattern that differs markedly from estrogen-induced subcutaneous fat accumulation. Visceral fat is characterized by high metabolic activity and drains directly into the liver via the portal vein. This anatomical and functional feature amplifies the hepatic metabolic burden of drugs, leading to increased drug clearance rates in certain scenarios—thereby further exacerbating sex-specific disparities in systemic drug exposure and subsequent therapeutic responses.

### Drug-metabolizing enzymes: sex-biased activity driven by hormones

Sex differences in the activity of hepatic drug-metabolizing enzymes—particularly those of the CYP450 superfamily—are primarily mediated by sex hormones, with minimal influence from lifestyle factors. For instance, estrogen upregulates the activity of CYP3A4, a key member of the CYP450 family that plays a critical role in the metabolism of clinically relevant chemotherapeutics (e.g., docetaxel, vinorelbine). This estrogen-driven upregulation enhances CYP3A4-mediated drug clearance in women relative to men, which in turn reduces the concentration of active drug at tumor sites and may ultimately diminish therapeutic efficacy. Notably, such disparities in enzyme activity are consistent across diverse ethnic and demographic populations, underscoring their role as a universal biological determinant of sex-specific differences in treatment responses.

Collectively, sex-based disparities in treatment efficacy arise from three interconnected mechanisms: (1) the direct effects of sex hormones on tumor cell function and the development of drug resistance; (2) hormone-induced biases in anti-tumor immune responses; and (3) physiological differences (e.g., fat distribution, drug-metabolizing enzyme activity) that modulate drug pharmacokinetics and pharmacodynamics. Addressing these disparities thus necessitates sex-tailored therapeutic strategies. For example, dose adjustments for lipophilic drugs could be guided by sex-specific fat distribution patterns to optimize drug exposure; alternatively, targeting hormone-regulated drug-metabolizing enzymes (e.g., inhibiting estrogen-driven CYP3A4 overactivity in women) may help standardize the concentration of active drug at tumor sites. Additionally, administering exogenous hormone modulators to normalize systemic hormone levels may mitigate sex-related treatment disparities. However, this strategy requires rigorous clinical validation to ensure a balance between therapeutic efficacy and potential safety risks, as hormone modulation may introduce off-target effects in non-tumor tissues [134].

## Future directions: role of gut microbiota in sex differences in cancer

Growing evidence indicates that gut microbiota plays a pivotal role in mediating sex differences in tumor progression and treatment outcomes [135]. Sex-specific microbial profiles are shaped by the interplay of genetic factors and sex hormones, with premenopausal women generally exhibiting higher microbial diversity and greater abundance of beneficial taxa (e.g., Bifidobacterium) than men [135, 136]. Sex steroids can directly reshape gut microbial communities by altering nutrient substrates and microbial enzyme activity [137], and indirectly via host physiological functions such as motility, hormone secretion, and mucosal immunity [136, 138–140]. In castration-resistant prostate cancer (CRPC), symbiotic gut bacteria further fuel tumor growth through de novo androgen synthesis, providing an alternative hormonal source to sustain AR-driven oncogenic signaling [141]. The gut microbiota also modulates anti-tumor immunity: for instance, it drives eosinophil recruitment by promoting Th17 differentiation and IL-17 secretion, accelerating multiple myeloma progression [142]; it can also enhance the efficacy of chemotherapeutic agents (e.g., cyclophosphamide) and immune checkpoint inhibitors (e.g., anti-PD-L1) via T-cell regulation [143, 144].

Despite these advances, current research remains largely confined to colorectal and prostate cancers, with limited cross-cancer validation, and key mechanisms of the “sex hormone–gut microbiota–tumor” axis are unresolved—for example, how sex hormones precisely modulate microbiota composition to influence tumor progression, and the contribution of microbiota-derived metabolites such as lysophosphatidylcholine [135]. Under physiological conditions, the greater abundance of beneficial bacteria in the female gut may confer protection against tumor initiation. Following malignant transformation, gut microbes impact tumor cells directly (via metabolites, cytokines, or microbial hormones) and indirectly (via modulation of anti-tumor immunity). Hormone-induced sex differences in gut microbiota likely exert systemic effects beyond the gut, potentially driving sex disparities across cancers—particularly hormone-independent ones like CRC. These insights motivate translational exploration of sex-specific microbiome interventions; for example, male-targeted Lactobacillus-based formulations could be tested to improve prognosis in gut-associated cancers.

Moving forward, future studies should utilize multi-omics analyses (metagenomics, metabolomics, host transcriptomics) in large, sex-stratified patient cohorts, combined with longitudinal follow-up, to establish causal relationships and identify predictive microbial biomarkers. Preclinical models should investigate whether interventions such as sex-specific probiotics, dietary modulation, or microbiota transplantation can enhance cancer treatment efficacy, as early evidence suggests male-targeted Lactobacillus-based formulations may improve outcomes in gut-associated cancers. Clarifying these mechanisms will open new avenues for precision oncology strategies that integrate sex differences and microbiome modulation.

## Discussion

Sexual dimorphism in cancer arises from the interplay of sex chromosome-linked genes, epigenetic regulation, hormonal signaling, metabolic programming, immune modulation, and stromal cell biology. While sex hormones are prominent drivers—especially in reproductive organ cancers—a balanced interpretation requires integrating non-hormonal mechanisms and system-level effects to fully explain sex differences across diverse malignancies.

Under physiological conditions, estrogen contributes to tissue homeostasis and supports aspects of immune function. However, in hormone-dependent tumors such as breast, ovarian, and endometrial cancers, elevated estrogen signaling can promote tumor progression and correlate with poorer outcomes. In certain hormone-independent malignancies, estrogen exhibits context-dependent effects; for example, in lung cancer, it may facilitate malignant progression, yet observational data suggest potential protective associations in hormone replacement therapy under specific conditions [145]. These dual effects underscore the need to understand dose, timing, receptor isoforms, and tissue context when evaluating estrogen’s role.

Androgens and the AR are critical in prostate cancer but also influence biology in malignancies not traditionally considered hormone-driven. AR can intersect with other hormonal and growth factor pathways, modulate metabolic programs, and shape the tumor microenvironment. Emerging evidence of AR pathway activity outside the prostate highlights the importance of studying how estrogen–AR–other endocrine signals collectively contribute to sex-biased tumor phenotypes and treatment responses.

Beyond direct tumor cell signaling, hormones interact with the gut microbiota to exert systemic effects on metabolism and immunity, thereby influencing tumor initiation, progression, and therapy response. Although much of the current evidence derives from gastrointestinal cancers, sex-hormone–microbiota interactions are increasingly recognized as relevant across non-GI malignancies. Importantly, hormone-induced shifts in microbiota composition and function can modulate drug metabolism and anti-tumor immune tone, offering testable targets for sex-informed interventions.

Methodologically, future work should move beyond isolated in vitro models toward integrative in vivo and ex vivo systems that capture the ecological complexity of the tumor microenvironment, including simultaneous changes in stromal, immune, and vascular compartments and compensatory endocrine effects. Large, sex-stratified, multi-omics cohorts with longitudinal sampling are needed to establish causal links across mechanisms and to identify biomarkers that predict sex-specific risk and therapeutic benefit.

Precision oncology provides a pathway to translate these mechanistic insights into tangible clinical benefit.

First, biomarker development is key: sex-stratified panels that integrate metabolic signatures, immune phenotypes (e.g., T-cell exhaustion markers, cytokine profiles), and microbiome-derived metabolites could enhance early detection, refine prognostic models, and guide individualized therapy selection.

Second, therapeutic optimization should leverage sex-aware strategies. This may involve tailoring immunotherapy regimens according to documented differences in response between men and women, integrating metabolic inhibitors to offset male-biased glycolytic or lipogenic programs, and adjusting dosing schedules based on sex-related pharmacokinetic and pharmacodynamic profiles. Targeted modulation of drug-metabolizing enzymes—such as correcting estrogen‑driven CYP3A4 overactivity—could normalize active drug concentrations at tumor sites, while exogenous hormone modulators may help mitigate sex‑related disparities in treatment efficacy; such interventions require rigorous clinical validation to balance benefit with safety.

Third, clinical trial design must evolve to capture sex effects accurately. Prospective trials should pre-specify sex-stratified endpoints, be powered to detect interactions by sex, and ensure standardized reporting of sex-specific adverse events, PK/PD parameters, and quality-of-life measures. Likewise, real‑world registries should consistently collect and analyze sex-stratified data to inform evidence-based updates to national guidelines. Current knowledge gaps include limited cross-cancer validation of hormone–microbiota mechanisms, epigenetic regulation in sex-difference research, and insufficient integration of stromal and vascular biology. Addressing these gaps will refine mechanistic models and support rational, sex-informed clinical decision-making.

Finally, ongoing and registered clinical studies examining sex differences in epidemiology, treatment efficacy, toxicity, and psychosocial outcomes will be instrumental in translating research into practice (Table 2). Aligning these efforts with national guideline development and policy frameworks will help ensure equitable prevention, diagnosis, and treatment—advancing a precision oncology paradigm attuned to biological sex.

**Table 2:** Clinical trials on effect of sex difference in tumor

No.	Cancer Type	Country	Status	Enrollment	Study Methods	Study Type	Study Purpose	Register Information	
**Epidemiology**									
1	Gastric Cancer	Korea	Completed	14,900 (Actual)	Observational	Retrospective Study	Sex Difference with Incidence of Histologic Subtypes and Survival Rate	NCT04973631	
2	Genitourinary Cancers	United States	Recruiting	80 (Estimated)	Observational	Prospective Study	Gender Related Coping and Survivorship	NCT05649306	
**Tumor Treatment**									
3	Bladder cancer	Italy	Unknown status	500 (Estimated)	Observational	Retrospective Study	Gender-related Characteristics of Bladder Cancer Treatment	NCT04687254	
4	Pan-Cancer	Italy	Completed	247 (Actual)	Observational	Multicenter Prospective Study	Gender Difference in side effects of Immunotherapy	NCT04435964	
5	Hepatocellular Carcinoma	China	Completed	3769 (Actual)	Observational	Multicenter Retrospective Study	Impact of Gender on the Outcome of Liver Transplantation for Hepatocellular Carcinoma	NCT05643833	
6	Pediatric Brain Tumor	United States	Terminated	2 (Actual)	Interventional	Prospective Study	Therapeutic Targeting of Sex Differences in Pediatric Brain Tumor Glycolysis	NCT03591861	
7	Differentiated Thyroid Cancer	Italy	Recruiting	50 (Estimated)	Observational	Prospective Study	Gender-based Impact on Safety and Efficacy of Lenvatinib	NCT05789667	
**Social Psychology**									
8	Pan-Cancer	Italy	Recruiting	500 (Estimated)	Observational	Prospective Study	Gender Difference with Distress and Fatigue	NCT05122052	
9	Advanced Non-small Cell Lung Cancer	Spain	Terminated	344 (Actual)	Observational	Multicenter Prospective study	Gender Differences on the Psychosocial and Economic Impact	NCT02336061	

## Conclusion

**Fig. 3:**
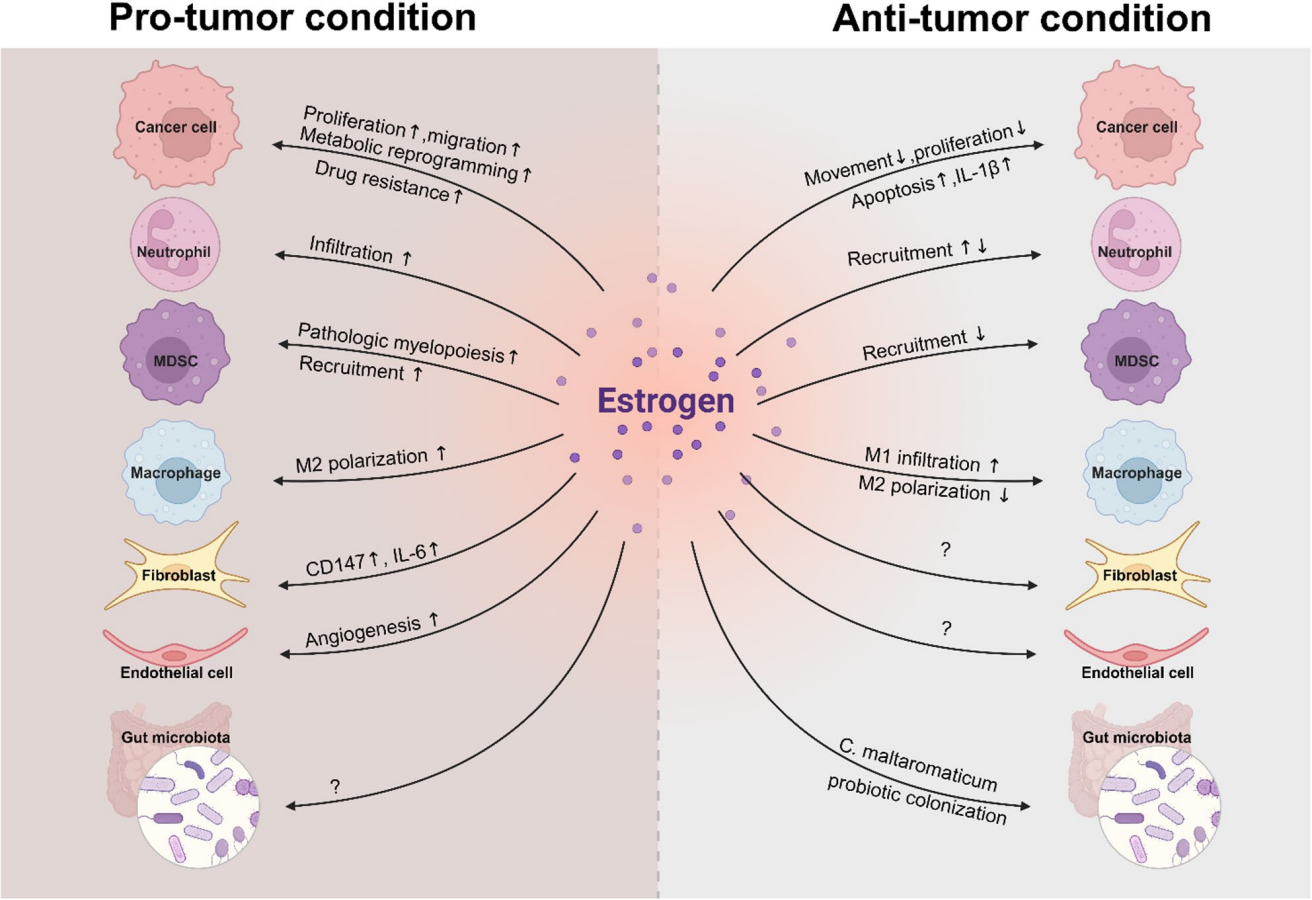
Complex and contradictory roles of estrogen in tumor development

In this review, we systematically summarized sex disparities in cancer epidemiology and delineated the key pathways and determinants underlying sex-related differences in cancer incidence and prognosis. We dissected how sex differences modulate tumor initiation and progression from six interconnected dimensions: sex chromosomes and genetics, tumor cells, immunity, tumor stromal cells, and therapeutic responses. Additionally, we highlighted the current limitations and unmet research needs in these areas, which lays a theoretical foundation for optimizing tumor treatment efficacy in both males and females and mitigating sex-related disparities in cancer treatment outcomes (Fig. 3). Estrogen and androgens emerge as core regulators of cancer sex differences—estrogen predominates in female-predominant malignancies (e.g., breast, endometrial cancer), whereas androgens play a pivotal role in male-predominant cancers (e.g., prostate, bladder cancer). Both hormones exert context-dependent bidirectional effects—with their actions contingent on tumor type—and their crosstalk further modulates sex-specific cancer trajectories.

The impact of estrogen on specific cells varies depending on the tumor and can even have opposing effects. The figure shows that both pro-tumor and anti-tumor effects of estrogen have been reported on tumor cells, neutrophils, MDSCs, and macrophages. It is reasonable to speculate that there are also bi-directional effects of estrogen on lymphocytes, fibroblasts, endothelial cells, and intestinal flora. This difference may be related to the subtype of hormone receptor expression. However, it is still unknown and requires further exploration.

## Data Availability

The data generated in this study are available within the article and its supplementary data files.

## Abbreviations

AP-1: Activator protein 1
AR: Androgen receptor
AREs: Androgen response elements
BFGF: Basic fibroblast growth factor
BMI: Body mass index
CAFs: Cancer-associated fibroblasts
CDK6: Cyclin-dependent kinase 6
CNS: Central nervous system
CRC: Colorectal cancer
DNMT1: DNA methyltransferase 1
ECM: Extracellular matrix
ECP: Estradiol cypionate
ER: Estrogen receptors
ERE: Estrogen response element
EVs: Extracellular vesicles
FN1: Fibronectin 1
HCCs: Hepatocellular carcinomas
HIF-1: Hypoxia inducible factor-1
HUVECs: Human umbilical vein endothelial cells
ICB: Immune checkpoint blockade
LncRNAs: Long noncoding RNAs
LOY: Loss of the Y chromosome
MAPK: Mitogen-activated protein kinase
MaSCs: Mammary stem cells
MDSCs: Myeloid-derived suppressor cells
MISS: Membrane-initiated steroid signaling
MMPs: Matrix metalloproteinases
MSCs: Mesenchymal stromal cells
NISS: Nuclear-initiated steroid signaling
NSCLC: Non-small cell lung cancer
PA: Plasminogen activator
PCa: Prostate cancer
PI3K: Phosphatidylinositol-3 kinase
S1P: Sphingosine 1-phosphate
SEER: Surveillance, Epidemiology, and End Results
SP-1: Specificity protein 1
TME: Tumor microenvironment
TSPY: Testis-specific protein Y-encoded protein
UC: Urothelial cancer
VEGF: Vascular endothelial growth factor
XCI: X chromosome inactivation
Xist: X inactive-specific transcript

## References

[1] Siegel RL, Kratzer TB, Giaquinto AN, Sung H, Jemal A. Cancer statistics, 2025. CA Cancer J Clin. 2025;75(1):10–45. 10.3322/caac.21871. 39817679 10.3322/caac.21871 PMC11745215

[2] Fu Y, Liu J, Chen Y, Liu Z, Xia H, Xu H. Gender disparities in lung cancer incidence in the United States during 2001–2019. Sci Rep. 2023;13(1):12581. 10.1038/s41598-023-39440-8. 37537259 10.1038/s41598-023-39440-8 PMC10400573

[3] Keum N, Giovannucci E. Global burden of colorectal cancer: emerging trends, risk factors and prevention strategies. Nat Rev Gastroenterol Hepatol. 2019;16:12. 10.1038/s41575-019-0189-8. 10.1038/s41575-019-0189-8 31455888

[4] Li Q, Lu Y, Wang D. Differential survival outcomes and prognostic factors across surgical approaches for colorectal cancer: analysis of 5000 patients from the SEER database. Curr Probl Surg. 2025;70:101839. 10.1016/j.cpsurg.2025.101839. 40812984 10.1016/j.cpsurg.2025.101839

[5] Costa AR, de Lança Oliveira M, Cruz I, Gonçalves I, Cascalheira JF, Santos CRA. The sex bias of cancer. Trends Endocrinol Metab. 2020;31(10):785–99. 10.1016/j.tem.2020.07.002. 32900596 10.1016/j.tem.2020.07.002

[6] Jackson SS, Marks MA, Katki HA, Cook MB, Hyun N, Freedman ND, et al. Sex disparities in the incidence of 21 cancer types: quantification of the contribution of risk factors. Cancer. 2022;128(19):3531–40. 10.1002/cncr.34390. 35934938 10.1002/cncr.34390 PMC11578066

[7] Cook MB, McGlynn KA, Devesa SS, Freedman ND, Anderson WF. Sex disparities in cancer mortality and survival. Cancer Epidemiol Biomarkers Prev. 2011;20(8):1629–37. 10.1158/1055-9965.Epi-11-0246. 21750167 10.1158/1055-9965.EPI-11-0246 PMC3153584

[8] Jeon DS, Kim JW, Kim SG, Kim HR, Song SY, Lee JC, et al. Sex differences in the characteristics and survival of patients with non-small-cell lung cancer: a retrospective analytical study based on real-world clinical data of the Korean population. Thorac Cancer. 2022;13(18):2584–91. 10.1111/1759-7714.14594. 35906163 10.1111/1759-7714.14594 PMC9475225

[9] Buja A, Rugge M, Damiani G, Zorzi M, De Toni C, Vecchiato A, et al. Sex differences in cutaneous melanoma: incidence, clinicopathological profile, survival, and costs. J Womens Health. 2022;31(7):1012–9. 10.1089/jwh.2021.0223. 10.1089/jwh.2021.0223 PMC9299528 35076310

[10] Senft D. Sex affects cancer genomes. Nat Rev Cancer. 2024;24:2. 10.1038/s41568-024-00663-0. 38200132 10.1038/s41568-024-00663-0

[11] Ozga M, Nicolet D, Mrózek K, Yilmaz AS, Kohlschmidt J, Larkin KT, et al. Sex-associated differences in frequencies and prognostic impact of recurrent genetic alterations in adult acute myeloid leukemia (Alliance, AMLCG). Leukemia. 2024;38(1):45–57. 10.1038/s41375-023-02068-8. 38017103 10.1038/s41375-023-02068-8 PMC10776397

[12] Brouwer AF, Engle JM, Jeon J, Meza R. Sociodemographic survival disparities for lung cancer in the united States, 2000–2016. J Natl Cancer Inst. 2022;114:111492–500. 10.1093/jnci/djac144. 10.1093/jnci/djac144 PMC9664170 35866998

[13] Richters A, Aben KKH, Kiemeney L. The global burden of urinary bladder cancer: an update. World J Urol. 2020;38(8):1895–904. 10.1007/s00345-019-02984-4. 31676912 10.1007/s00345-019-02984-4 PMC7363726

[14] Rumgay H, Ferlay J, de Martel C, Georges D, Ibrahim AS, Zheng R, et al. Global, regional and national burden of primary liver cancer by subtype. Eur J Cancer. 2022;161:108–18. 10.1016/j.ejca.2021.11.023. 34942552 10.1016/j.ejca.2021.11.023

[15] Islami F, Miller KD, Siegel RL, Fedewa SA, Ward EM, Jemal A. Disparities in liver cancer occurrence in the united States by race/ethnicity and state. CA Cancer J Clin. 2017;67:4273–89. 10.3322/caac.21402. 10.3322/caac.21402 28586094

[16] Schafer EJ, Jemal A, Wiese D, Sung H, Kratzer TB, Islami F, et al. Disparities and trends in genitourinary cancer incidence and mortality in the USA. Eur Urol. 2023;84(1):117–26. 10.1016/j.eururo.2022.11.023. 36566154 10.1016/j.eururo.2022.11.023

[17] Song M, Kang D, Yang JJ, Choi JY, Sung H, Lee Y, et al. Age and sex interactions in gastric cancer incidence and mortality trends in Korea. Gastric Cancer. 2015;18(3):580–9. 10.1007/s10120-014-0411-x. 25091081 10.1007/s10120-014-0411-x

[18] Zhou J, Zheng R, Zhang S, Chen R, Wang S, Sun K, et al. Gastric and esophageal cancer in China 2000 to 2030: recent trends and short-term predictions of the future burden. Cancer Med. 2022;11(8):1902–12. 10.1002/cam4.4586. 35148032 10.1002/cam4.4586 PMC9041080

[19] Haupt S, Caramia F, Klein SL, Rubin JB, Haupt Y. Sex disparities matter in cancer development and therapy. Nat Rev Cancer. 2021;21(6):393–407. 10.1038/s41568-021-00348-y. 33879867 10.1038/s41568-021-00348-y PMC8284191

[20] Lopes-Ramos CM, Quackenbush J, DeMeo DL. Genome-wide sex and gender differences in cancer. Front Oncol. 2020;10:597788. 10.3389/fonc.2020.597788. 33330090 10.3389/fonc.2020.597788 PMC7719817

[21] Dunford A, Weinstock DM, Savova V, Schumacher SE, Cleary JP, Yoda A, et al. Tumor-suppressor genes that escape from X-inactivation contribute to cancer sex bias. Nat Genet. 2017;49(1):10–6. 10.1038/ng.3726. 27869828 10.1038/ng.3726 PMC5206905

[22] Navarro-Cobos MJ, Balaton BP, Brown CJ. Genes that escape from X-chromosome inactivation: potential contributors to Klinefelter syndrome. Am J Med Genet C Semin Med Genet. 2020;184(2):226–38. 10.1002/ajmg.c.31800. 32441398 10.1002/ajmg.c.31800 PMC7384012

[23] Wang D, Tang L, Wu Y, Fan C, Zhang S, Xiang B, et al. Abnormal X chromosome inactivation and tumor development. Cell Mol Life Sci. 2020;77(15):2949–58. 10.1007/s00018-020-03469-z. 32040694 10.1007/s00018-020-03469-z PMC11104905

[24] Trotman JB, Calabrese JM. How to silence an X chromosome. Nature. 2020;578(7795):365–6. 10.1038/d41586-020-00207-0. 32066915 10.1038/d41586-020-00207-0

[25] Richart L, Picod-Chedotel ML, Wassef M, Macario M, Aflaki S, Salvador MA, et al. XIST loss impairs mammary stem cell differentiation and increases tumorigenicity through mediator hyperactivation. Cell. 2022;185(12):2164–e8325. 10.1016/j.cell.2022.04.034. 35597241 10.1016/j.cell.2022.04.034

[26] Yang X, Zhang S, He C, Xue P, Zhang L, He Z, et al. METTL14 suppresses proliferation and metastasis of colorectal cancer by down-regulating oncogenic long non-coding RNA XIST. Mol Cancer. 2020;19(1):46. 10.1186/s12943-020-1146-4. 32111213 10.1186/s12943-020-1146-4 PMC7047419

[27] Xiao G, Yao J, Kong D, Ye C, Chen R, Li L, et al. The long noncoding RNA TTTY15, which is located on the Y chromosome, promotes prostate cancer progression by sponging let-7. Eur Urol. 2019;76(3):315–26. 10.1016/j.eururo.2018.11.012. 30527798 10.1016/j.eururo.2018.11.012

[28] Kido T, Lau YC. The Y-linked proto-oncogene TSPY contributes to poor prognosis of the male hepatocellular carcinoma patients by promoting the pro-oncogenic and suppressing the anti-oncogenic gene expression. Cell Biosci. 2019;9:22. 10.1186/s13578-019-0287-x. 30867900 10.1186/s13578-019-0287-x PMC6399826

[29] Li J, Lan Z, Liao W, Horner JW, Xu X, Liu J, et al. Histone demethylase KDM5D upregulation drives sex differences in colon cancer. Nature. 2023;619(7970):632–9. 10.1038/s41586-023-06254-7. 37344599 10.1038/s41586-023-06254-7 PMC10529424

[30] Cáceres A, Jene A, Esko T, Pérez-Jurado LA, González JR. Extreme downregulation of chromosome Y and cancer risk in men. J Natl Cancer Inst. 2020;112(9):913–20. 10.1093/jnci/djz232. 31945786 10.1093/jnci/djz232 PMC7492764

[31] Abdel-Hafiz HA, Schafer JM, Chen X, Xiao T, Gauntner TD, Li Z, et al. Y chromosome loss in cancer drives growth by evasion of adaptive immunity. Nature. 2023;619(7970):624–31. 10.1038/s41586-023-06234-x. 37344596 10.1038/s41586-023-06234-x PMC10975863

[32] Solomon O, Huen K, Yousefi P, Küpers LK, González JR, Suderman M, et al. Meta-analysis of epigenome-wide association studies in newborns and children show widespread sex differences in blood DNA methylation. Mutation Research/Reviews in Mutation Research. 2022;789:108415. 10.1016/j.mrrev.2022.108415. 35690418 10.1016/j.mrrev.2022.108415 PMC9623595

[33] Tian T, Bi H, Zhang D, Liu Y, Sun H, Jia C, et al. Methylation of three genes encoded by X chromosome in blood leukocytes and colorectal cancer risk. Cancer Med. 2021;10(14):4964–76. 10.1002/cam4.4056. 34145793 10.1002/cam4.4056 PMC8290255

[34] Wang J, Huang F, Huang J, Kong J, Liu S, Jin J. Epigenetic analysis of FHL1 tumor suppressor gene in human liver cancer. Oncol Lett. 2017;14(5):6109–16. 10.3892/ol.2017.6950. 29113254 10.3892/ol.2017.6950 PMC5661399

[35] Lam CM, Li Z, Theodorescu D, Li X. Mechanism of sex differences in bladder cancer: evident and elusive sex-biasing factors. Bladder Cancer. 2022;8:3241–54. 10.3233/blc-211658. 10.3233/BLC-211658 PMC9536425 36277328

[36] Zhou J, Li M, Chen Y, Wang S, Wang D, Suo C, et al. Attenuated sex-related DNA methylation differences in cancer highlight the magnitude bias mediating existing disparities. Biol Sex Differ. 2024;15(1):106. 10.1186/s13293-024-00682-4. 39716176 10.1186/s13293-024-00682-4 PMC11664931

[37] Kiesel B, Borkovec M, Furtner J, Roetzer-Pejrimovsky T, Nenning KH, Greutter L, et al. Sex-specific differences in DNA methylation defining prognostically relevant subgroups in glioblastoma. J Neurosurg. 2025;143(1):204–13. 10.3171/2024.9.Jns24665. 39951709 10.3171/2024.9.JNS24665

[38] Willis-Owen SAG, Domingo-Sabugo C, Starren E, Liang L, Freidin MB, Arseneault M, et al. Y disruption, autosomal hypomethylation and poor male lung cancer survival. Sci Rep. 2021;11(1):12453. 10.1038/s41598-021-91907-8. 34127738 10.1038/s41598-021-91907-8 PMC8203787

[39] Lin TS, Lee H, Chen RA, Ho ML, Lin CY, Chen YH, et al. An association of DNMT3b protein expression with P16INK4a promoter hypermethylation in non-smoking female lung cancer with human papillomavirus infection. Cancer Lett. 2005;226(1):77–84. 10.1016/j.canlet.2004.12.031. 16004934 10.1016/j.canlet.2004.12.031

[40] Xiao Y, Word B, Starlard-Davenport A, Haefele A, Lyn-Cook BD, Hammons G. Age and gender affect DNMT3a and DNMT3b expression in human liver. Cell Biol Toxicol. 2008;24(3):265–72. 10.1007/s10565-007-9035-9. 17929180 10.1007/s10565-007-9035-9

[41] Nugent BM, Wright CL, Shetty AC, Hodes GE, Lenz KM, Mahurkar A, et al. Brain feminization requires active repression of masculinization via DNA methylation. Nat Neurosci. 2015;18(5):690–7. 10.1038/nn.3988. 25821913 10.1038/nn.3988 PMC4519828

[42] Mitchnick KA, Nicholson K, Wideman C, Jardine K, Jamieson-Williams R, Creighton SD, et al. The lysine acetyltransferase PCAF functionally interacts with estrogen receptor alpha in the hippocampus of gonadally intact male-but not female-rats to enhance short-term memory. J Neurosci. 2024. 10.1523/jneurosci.1574-23.2024. 39138001 10.1523/JNEUROSCI.1574-23.2024 PMC11376336

[43] Nazário WR, Costa A, de Oliveira Santos D, Del Nero NRD, Rodrigues TS, Borges LDF, et al. Sex-Related differences in histone acetylation and tumor development in a 4-Nitroquinoline 1-Oxide and Ethanol-Induced oral squamous cell carcinoma mouse model. J Oral Pathol Med. 2025;54:10:1074–84. 10.1111/jop.70062. 40928052 10.1111/jop.70062 PMC12602132

[44] Nguyen T, Sridaran D, Chouhan S, Weimholt C, Wilson A, Luo J, et al. Histone H2A lys130 acetylation epigenetically regulates androgen production in prostate cancer. Nat Commun. 2023;14(1):3357. 10.1038/s41467-023-38887-7. 37296155 10.1038/s41467-023-38887-7 PMC10256812

[45] Anguera MC, Sadreyev R, Zhang Z, Szanto A, Payer B, Sheridan SD, et al. Molecular signatures of human induced pluripotent stem cells highlight sex differences and cancer genes. Cell Stem Cell. 2012;11(1):75–90. 10.1016/j.stem.2012.03.008. 22770242 10.1016/j.stem.2012.03.008 PMC3587778

[46] Severson TM, Minnee E, Zhu Y, Schuurman K, Nguyen HM, Brown LG, et al. Epigenetic profiling identifies markers of endocrine resistance and therapeutic options for metastatic castration-resistant prostate cancer. Cell Rep Med. 2025;6(7):102215. 10.1016/j.xcrm.2025.102215. 40609538 10.1016/j.xcrm.2025.102215 PMC12281433

[47] Lasagna M, Enriquez L, Mardirosian M, Cardozo RN, Martín G, Power P, et al. Chlorpyrifos and tamoxifen co-pretreatment promotes stem-like phenotype and upregulation of anti-estrogen therapy resistance markers in ERα breast cancer cells. Environ Res. 2026;290:123447. 10.1016/j.envres.2025.123447. 41319740 10.1016/j.envres.2025.123447

[48] Kaur P, Verma S, Kushwaha PP, Gupta S. EZH2 and NF-κB: a context-dependent crosstalk and transcriptional regulation in cancer. Cancer Lett. 2023;560:216143. 10.1016/j.canlet.2023.216143. 36958695 10.1016/j.canlet.2023.216143

[49] Venkadakrishnan VB, Presser AG, Singh R, Booker MA, Traphagen NA, Weng K, et al. Lineage-specific canonical and non-canonical activity of EZH2 in advanced prostate cancer subtypes. Nat Commun. 2024;15(1):6779. 10.1038/s41467-024-51156-5. 39117665 10.1038/s41467-024-51156-5 PMC11310309

[50] Chhabra Y, Fane ME, Pramod S, Hüser L, Zabransky DJ, Wang V, et al. Sex-dependent effects in the aged melanoma tumor microenvironment influence invasion and resistance to targeted therapy. Cell. 2024;187:21. 10.1016/j.cell.2024.08.013. 10.1016/j.cell.2024.08.013 PMC11580838 39243764

[51] Tricarico R, Nicolas E, Hall MJ, Golemis EA. X- and Y-Linked chromatin-modifying genes as regulators of sex-specific cancer incidence and prognosis. Clin Cancer Res. 2020;26(21):5567–78. 10.1158/1078-0432.Ccr-20-1741. 32732223 10.1158/1078-0432.CCR-20-1741 PMC7642178

[52] Kaneko S, Li X. X chromosome protects against bladder cancer in females via a KDM6A-dependent epigenetic mechanism. Sci Adv. 2018;4(6):eaar5598. 10.1126/sciadv.aar5598. 29928692 10.1126/sciadv.aar5598 PMC6007159

[53] Tian JM, Dong YH, Li Z, Zhou Y, Huang J. Androgen-induced AR-BRD4 transcriptional regulatory complex promotes malignant proliferation of osteosarcoma cells. Cell Death Discov. 2025;11(1):272. 10.1038/s41420-025-02541-6. 40494882 10.1038/s41420-025-02541-6 PMC12152148

[54] Murakami S, Li R, Nagari A, Chae M, Camacho CV, Kraus WL. Distinct roles for BET family members in estrogen receptor α enhancer function and gene regulation in breast cancer cells. Mol Cancer Res. 2019;17(12):2356–68. 10.1158/1541-7786.Mcr-19-0393. 31551256 10.1158/1541-7786.MCR-19-0393 PMC6891197

[55] Nagarajan S, Hossan T, Alawi M, Najafova Z, Indenbirken D, Bedi U, et al. Bromodomain protein BRD4 is required for estrogen receptor-dependent enhancer activation and gene transcription. Cell Rep. 2014;8(2):460–9. 10.1016/j.celrep.2014.06.016. 25017071 10.1016/j.celrep.2014.06.016 PMC4747248

[56] Carè A, Bellenghi M, Matarrese P, Gabriele L, Salvioli S, Malorni W. Sex disparity in cancer: roles of microRNAs and related functional players. Cell Death Differ. 2018;25(3):477–85. 10.1038/s41418-017-0051-x. 29352271 10.1038/s41418-017-0051-x PMC5864217

[57] Huerne K, Jackson SS, Lall R, Palmour N, Berner AM, Dupras C, et al. Studies in cancer epigenetics through a sex and gendered lens: a comprehensive scoping review. Cancers (Basel). 2023. 10.3390/cancers15174207. 37686484 10.3390/cancers15174207 PMC10486657

[58] Bellenghi M, Puglisi R, Pontecorvi G, De Feo A, Carè A, Mattia G. Sex and gender disparities in melanoma. Cancers (Basel). 2020. 10.3390/cancers12071819. 32645881 10.3390/cancers12071819 PMC7408637

[59] Tomeva E, Krammer UDB, Switzeny OJ, Haslberger AG, Hippe B. Sex-specific miRNA differences in liquid biopsies from subjects with solid tumors and healthy controls. Epigenomes. 2023. 10.3390/epigenomes7010002. 36648863 10.3390/epigenomes7010002 PMC9844450

[60] Chan KK, Au KY, Fung WC, Wong CY, Chan AC, Lo RC. Sex-specific analysis of microRNA profiles in HBV-associated cirrhosis by small RNA-sequencing. Hepatol Commun. 2022;6(12):3473–86. 10.1002/hep4.2096. 36166204 10.1002/hep4.2096 PMC9701490

[61] Zhao H, Feng L, Cheng R, Wu M, Bai X, Fan L, et al. miR-29c-3p acts as a tumor promoter by regulating β-catenin signaling through suppressing DNMT3A, TET1 and HBP1 in ovarian carcinoma. Cell Signal. 2024;113:110936. 10.1016/j.cellsig.2023.110936. 37925048 10.1016/j.cellsig.2023.110936

[62] Mazzu YZ, Yoshikawa Y, Nandakumar S, Chakraborty G, Armenia J, Jehane LE, et al. Methylation-associated miR-193b silencing activates master drivers of aggressive prostate cancer. Mol Oncol. 2019;13(9):1944–58. 10.1002/1878-0261.12536. 31225930 10.1002/1878-0261.12536 PMC6717747

[63] Zhong S, Borlak J. Sex differences in the tumor promoting effects of tobacco smoke in a cRaf transgenic lung cancer disease model. Arch Toxicol. 2024;98(3):957–83. 10.1007/s00204-023-03671-5. 38245882 10.1007/s00204-023-03671-5 PMC10861769

[64] Caserta S, Gangemi S, Murdaca G, Allegra A. Gender differences and miRNAs expression in cancer: implications on prognosis and susceptibility. Int J Mol Sci. 2023. 10.3390/ijms241411544. 37511303 10.3390/ijms241411544 PMC10380791

[65] Takeiwa T, Ikeda K, Horie-Inoue K, Inoue S. Mechanisms of apoptosis-related long non-coding RNAs in ovarian cancer. Front Cell Dev Biol. 2021;9:641963. 10.3389/fcell.2021.641963. 33996797 10.3389/fcell.2021.641963 PMC8117355

[66] Varajti K, Vereczkei A, Kovács-Valasek M, Zand A, Varjas T, Kiss I. An exploratory analysis of tumor site- and sex-specific associations of SNPs of LncRNA CCAT1, CCAT2, H19, HOTAIR, and PTCSC3 in colorectal lesions: a Hungarian case-control study. Biomedicines. 2025. 10.3390/biomedicines13123058. 41463069 10.3390/biomedicines13123058 PMC12731008

[67] Wang S, Pan W, Mi WX, Wang SH. Sex-specific gene expression patterns in head and neck squamous cell carcinomas. Heliyon. 2023;9(4):e14890. 10.1016/j.heliyon.2023.e14890. 37064442 10.1016/j.heliyon.2023.e14890 PMC10102211

[68] Liang J, Jin W, Xu H. An efficient five-lncRNA signature for lung adenocarcinoma prognosis, with AL606489.1 showing sexual dimorphism. Front Genet. 2022;13:1052092. 10.3389/fgene.2022.1052092. 36531243 10.3389/fgene.2022.1052092 PMC9748423

[69] Dioken DN, Ozgul I, Yilmazbilek I, Almeric E, Eroglu IC, Cicek M, et al. E2-regulated transcriptome complexity revealed by long-read direct RNA sequencing: from isoform discovery to truncated proteins. RNA Biol. 2025;22(1):1–21. 10.1080/15476286.2025.2563860. 40965441 10.1080/15476286.2025.2563860 PMC12456218

[70] Sanchez-Lopez JM, Juarez-Mancera MA, Bustamante B, Ruiz-Silvestre A, Espinosa M, Mendoza-Almanza G, et al. Decoding LINC00052 role in breast cancer by bioinformatic and experimental analyses. RNA Biol. 2024;21(1):1–11. 10.1080/15476286.2024.2355393. 38832821 10.1080/15476286.2024.2355393 PMC11152094

[71] Ferreira LB, Palumbo A, de Mello KD, Sternberg C, Caetano MS, de Oliveira FL, et al. PCA3 noncoding RNA is involved in the control of prostate-cancer cell survival and modulates androgen receptor signaling. BMC Cancer. 2012;12:507. 10.1186/1471-2407-12-507. 23130941 10.1186/1471-2407-12-507 PMC3544699

[72] Zhang Y, Pitchiaya S, Cieślik M, Niknafs YS, Tien JC, Hosono Y, et al. Analysis of the androgen receptor-regulated lncRNA landscape identifies a role for ARLNC1 in prostate cancer progression. Nat Genet. 2018;50(6):814–24. 10.1038/s41588-018-0120-1. 29808028 10.1038/s41588-018-0120-1 PMC5980762

[73] Xue X, Yang YA, Zhang A, Fong KW, Kim J, Song B, et al. LncRNA HOTAIR enhances ER signaling and confers tamoxifen resistance in breast cancer. Oncogene. 2016;35(21):2746–55. 10.1038/onc.2015.340. 26364613 10.1038/onc.2015.340 PMC4791209

[74] Gupta RA, Shah N, Wang KC, Kim J, Horlings HM, Wong DJ, et al. Long non-coding RNA HOTAIR reprograms chromatin state to promote cancer metastasis. Nature. 2010;464(7291):1071–6. 10.1038/nature08975. 20393566 10.1038/nature08975 PMC3049919

[75] Tao S, He H, Chen Q. Estradiol induces HOTAIR levels via GPER-mediated miR-148a inhibition in breast cancer. J Transl Med. 2015;13:131. 10.1186/s12967-015-0489-x. 25928008 10.1186/s12967-015-0489-x PMC4421993

[76] Yu W, Ding J, He M, Chen Y, Wang R, Han Z, et al. Estrogen receptor β promotes the vasculogenic mimicry (VM) and cell invasion via altering the lncRNA-MALAT1/miR-145-5p/NEDD9 signals in lung cancer. Oncogene. 2019;38(8):1225–38. 10.1038/s41388-018-0463-1. 30250297 10.1038/s41388-018-0463-1

[77] Schmidt K, Carroll JS, Yee E, Thomas DD, Wert-Lamas L, Neier SC, et al. The LncRNA SLNCR recruits the androgen receptor to EGR1-Bound genes in melanoma and inhibits expression of tumor suppressor p21. Cell Rep. 2019;27:82493–e5074. 10.1016/j.celrep.2019.04.101. 10.1016/j.celrep.2019.04.101 PMC6668037 31116991

[78] Zhai W, Sun Y, Guo C, Hu G, Wang M, Zheng J, et al. LncRNA-SARCC suppresses renal cell carcinoma (RCC) progression via altering the androgen receptor(AR)/miRNA-143-3p signals. Cell Death Differ. 2017;24(9):1502–17. 10.1038/cdd.2017.74. 28644440 10.1038/cdd.2017.74 PMC5563985

[79] Liu Y, Wang C, Li X, Dong L, Yang Q, Chen M, et al. Improved clinical outcome in a randomized phase II study of anti-PD-1 camrelizumab plus decitabine in relapsed/refractory Hodgkin lymphoma. J Immunother Cancer. 2021. 10.1136/jitc-2021-002347. 33820822 10.1136/jitc-2021-002347 PMC8025784

[80] Kumar RS, Goyal N. Estrogens as regulator of hematopoietic stem cell, immune cells and bone biology. Life Sci. 2021;269:119091. 10.1016/j.lfs.2021.119091. 33476629 10.1016/j.lfs.2021.119091

[81] Liu WJ, Zhao G, Zhang CY, Yang CQ, Zeng XB, Li J, et al. Comparison of the roles of estrogens and androgens in breast cancer and prostate cancer. J Cell Biochem. 2020;121(4):2756–69. 10.1002/jcb.29515. 31693255 10.1002/jcb.29515

[82] Mahboobifard F, Pourgholami MH, Jorjani M, Dargahi L, Amiri M, Sadeghi S, et al. Estrogen as a key regulator of energy homeostasis and metabolic health. Biomed Pharmacother. 2022;156:113808. 10.1016/j.biopha.2022.113808. 36252357 10.1016/j.biopha.2022.113808

[83] Samavat H, Kurzer MS. Estrogen metabolism and breast cancer. Cancer Lett. 2015;356(2 Pt A):231–43. 10.1016/j.canlet.2014.04.018. 24784887 10.1016/j.canlet.2014.04.018 PMC4505810

[84] Roepke TA, Qiu J, Bosch MA, Rønnekleiv OK, Kelly MJ. Cross-talk between membrane-initiated and nuclear-initiated oestrogen signalling in the hypothalamus. J Neuroendocrinol. 2009;21(4):263–70. 10.1111/j.1365-2826.2009.01846.x. 19187465 10.1111/j.1365-2826.2009.01846.x PMC2796511

[85] Roepke TA, Ronnekleiv OK, Kelly MJ. Physiological consequences of membrane-initiated estrogen signaling in the brain. Front Biosci (Landmark Ed). 2011;16(4):1560–73. 10.2741/3805. 21196248 10.2741/3805 PMC3094271

[86] Chen P, Li B, Ou-Yang L. Role of estrogen receptors in health and disease. Front Endocrinol (Lausanne). 2022;13:839005. 10.3389/fendo.2022.839005. 36060947 10.3389/fendo.2022.839005 PMC9433670

[87] Fish EN. The x-files in immunity: sex-based differences predispose immune responses. Nat Rev Immunol. 2008;8(9):737–44. 10.1038/nri2394. 18728636 10.1038/nri2394 PMC7097214

[88] Gubbels Bupp MR, Jorgensen TN. Androgen-induced immunosuppression. Front Immunol. 2018;9:794. 10.3389/fimmu.2018.00794. 29755457 10.3389/fimmu.2018.00794 PMC5932344

[89] Sciarra F, Campolo F, Franceschini E, Carlomagno F, Venneri MA. Gender-specific impact of sex hormones on the immune system. Int J Mol Sci. 2023. 10.3390/ijms24076302. 37047274 10.3390/ijms24076302 PMC10094624

[90] Grosse A, Bartsch S, Baniahmad A. Androgen receptor-mediated gene repression. Mol Cell Endocrinol. 2012;352(1–2):46–56. 10.1016/j.mce.2011.06.032. 21784131 10.1016/j.mce.2011.06.032

[91] Hsu HH, Kuo WW, Ju DT, Yeh YL, Tu CC, Tsai YL, et al. Estradiol agonists inhibit human LoVo colorectal-cancer cell proliferation and migration through p53. World J Gastroenterol. 2014;20:44:16665–73. 10.3748/wjg.v20.i44.16665. 25469035 10.3748/wjg.v20.i44.16665 PMC4248210

[92] Qiu X, Wang J, Zhang N, Du T, Chen L, Xi H. Estradiol cypionate inhibits proliferation and promotes apoptosis of gastric cancer by regulating AKT ubiquitination. Biomed Pharmacother. 2023;165:115073. 10.1016/j.biopha.2023.115073. 37392652 10.1016/j.biopha.2023.115073

[93] Zhao L, Huang S, Mei S, Yang Z, Xu L, Zhou N, et al. Pharmacological activation of estrogen receptor beta augments innate immunity to suppress cancer metastasis. Proc Natl Acad Sci U S A. 2018;115(16):E3673-e81. 10.1073/pnas.1803291115. 29592953 10.1073/pnas.1803291115 PMC5910874

[94] Yang F, Xie HY, Yang LF, Zhang L, Zhang FL, Liu HY, et al. Stabilization of MORC2 by estrogen and antiestrogens through GPER1- PRKACA-CMA pathway contributes to estrogen-induced proliferation and endocrine resistance of breast cancer cells. Autophagy. 2020;16(6):1061–76. 10.1080/15548627.2019.1659609. 32401166 10.1080/15548627.2019.1659609 PMC7469550

[95] Huang Q, Zhang Z, Liao Y, Liu C, Fan S, Wei X, et al. 17β-estradiol upregulates IL6 expression through the ERβ pathway to promote lung adenocarcinoma progression. J Exp Clin Cancer Res. 2018;37(1):133. 10.1186/s13046-018-0804-5. 29970138 10.1186/s13046-018-0804-5 PMC6029357

[96] Young MJ, Chen YC, Wang SA, Chang HP, Yang WB, Lee CC, et al. Estradiol-mediated inhibition of Sp1 decreases miR-3194-5p expression to enhance CD44 expression during lung cancer progression. J Biomed Sci. 2022;29(1):3. 10.1186/s12929-022-00787-1. 35034634 10.1186/s12929-022-00787-1 PMC8762881

[97] Zhang J, Song H, Lu Y, Chen H, Jiang S, Li L. Effects of estradiol on VEGF and bFGF by Akt in endometrial cancer cells are mediated through the NF-κB pathway. Oncol Rep. 2016;36:2705–14. 10.3892/or.2016.4888. 27349969 10.3892/or.2016.4888

[98] Lombardi APG, Vicente CM, Porto CS. Estrogen receptors promote migration, invasion and colony formation of the androgen-independent prostate cancer cells PC-3 through β-catenin pathway. Front Endocrinol (Lausanne). 2020;11:184. 10.3389/fendo.2020.00184. 32328032 10.3389/fendo.2020.00184 PMC7160699

[99] George AL, Rajoria S, Suriano R, Mittleman A, Tiwari RK. Hypoxia and estrogen are functionally equivalent in breast cancer-endothelial cell interdependence. Mol Cancer. 2012;11:80. 10.1186/1476-4598-11-80. 23088607 10.1186/1476-4598-11-80 PMC3504564

[100] Pacheco-Velázquez SC, Ortega M, II, Vargas-Navarro JL, Padilla-Flores JA, Robledo-Cadena DX, Tapia-Martínez G, et al. 17-β estradiol up-regulates energy metabolic pathways, cellular proliferation and tumor invasiveness in ER+ breast cancer spheroids. Front Oncol. 2022;12:1018137. 10.3389/fonc.2022.1018137. 36419896 10.3389/fonc.2022.1018137 PMC9676491

[101] Martinez-Bernabe T, Sastre-Serra J, Ciobu N, Oliver J, Pons DG, Roca P. Estrogen receptor beta (ERβ) maintains mitochondrial network regulating invasiveness in an obesity-related inflammation condition in breast cancer. Antioxidants. 2021. 10.3390/antiox10091371. 34573003 10.3390/antiox10091371 PMC8466315

[102] Riera Leal A, Ortiz-Lazareno PC, Jave-Suárez LF, Ramírez De Arellano A, Aguilar-Lemarroy A, Ortiz-García YM, et al. 17β‑estradiol‑induced mitochondrial dysfunction and Warburg effect in cervical cancer cells allow cell survival under metabolic stress. Int J Oncol. 2020;56(1):33–46. 10.3892/ijo.2019.4912. 31746421 10.3892/ijo.2019.4912 PMC6910176

[103] To SQ, Cheung V, Lazarus KA, Knower KC, Clyne CD. Estradiol regulates tumor necrosis factor-α expression and secretion in estrogen receptor positive breast cancer cells. Mol Cell Endocrinol. 2014;394:1–2. 10.1016/j.mce.2014.06.020. 25004254 10.1016/j.mce.2014.06.020

[104] Chen YC, Young MJ, Chang HP, Liu CY, Lee CC, Tseng YL, et al. Estradiol-mediated inhibition of DNMT1 decreases p53 expression to induce M2-macrophage polarization in lung cancer progression. Oncogenesis. 2022;11(1):25. 10.1038/s41389-022-00397-4. 35589688 10.1038/s41389-022-00397-4 PMC9119954

[105] Song S, Tang H, Quan W, Shang A, Ling C. Estradiol initiates the immune escape of non-small cell lung cancer cells via ERβ/SIRT1/FOXO3a/PD-L1 axis. Int Immunopharmacol. 2022;107:108629. 10.1016/j.intimp.2022.108629. 35344811 10.1016/j.intimp.2022.108629

[106] Drula R, Pardini B, Fu X, De Los Santos MC, Jurj A, Pang L, et al. 17β-estradiol promotes extracellular vesicle release and selective miRNA loading in ERα-positive breast cancer. Proc Natl Acad Sci U S A. 2023;120(23):e2122053120. 10.1073/pnas.2122053120. 37252969 10.1073/pnas.2122053120 PMC10266002

[107] Cavalieri E, Rogan E. The 3,4-quinones of estrone and estradiol are the initiators of cancer whereas resveratrol and N-acetylcysteine are the preventers. Int J Mol Sci. 2021. 10.3390/ijms22158238. 34361004 10.3390/ijms22158238 PMC8347442

[108] Takabe K, Kim RH, Allegood JC, Mitra P, Ramachandran S, Nagahashi M, et al. Estradiol induces export of sphingosine 1-phosphate from breast cancer cells via ABCC1 and ABCG2. J Biol Chem. 2010;285(14):10477–86. 10.1074/jbc.M109.064162. 20110355 10.1074/jbc.M109.064162 PMC2856255

[109] Chen X, Shen Y, Choi S, Abdel-Hafiz HA, Basu M, Hoelzen L, et al. Concurrent loss of the Y chromosome in cancer and T cells impacts outcome. Nature. 2025;642(8069):1041–50. 10.1038/s41586-025-09071-2. 40468066 10.1038/s41586-025-09071-2 PMC12221978

[110] Lee J, Nicosia M, Hong ES, Silver DJ, Li C, Bayik D, et al. Sex-biased T-cell exhaustion drives differential immune responses in glioblastoma. Cancer Discov. 2023;13(9):2090–105. 10.1158/2159-8290.Cd-22-0869. 37378557 10.1158/2159-8290.CD-22-0869 PMC10481130

[111] Cheng MI, Li JH, Riggan L, Chen B, Tafti RY, Chin S, et al. The X-linked epigenetic regulator UTX controls NK cell-intrinsic sex differences. Nat Immunol. 2023;24(5):780–91. 10.1038/s41590-023-01463-8. 36928413 10.1038/s41590-023-01463-8 PMC12882737

[112] Deng S, Ramos-Castaneda M, Velasco WV, Clowers MJ, Gutierrez BA, Noble O, et al. Interplay between estrogen and Stat3/NF-κB-driven immunomodulation in lung cancer. Carcinogenesis. 2020;41(11):1529–42. 10.1093/carcin/bgaa064. 32603404 10.1093/carcin/bgaa064 PMC7896112

[113] Song CH, Kim N, Nam RH, Choi SI, Jang JY, Kim JW, et al. Combination treatment with 17β-estradiol and anti-PD-L1 suppresses MC38 tumor growth by reducing PD-L1 expression and enhancing M1 macrophage population in MC38 colon tumor model. Cancer Lett. 2022;543:215780. 10.1016/j.canlet.2022.215780. 35690286 10.1016/j.canlet.2022.215780

[114] Chakraborty B, Byemerwa J, Shepherd J, Haines CN, Baldi R, Gong W, et al. Inhibition of estrogen signaling in myeloid cells increases tumor immunity in melanoma. J Clin Invest. 2021. 10.1172/jci151347. 34637400 10.1172/JCI151347 PMC8631601

[115] Shi Y, Pan J, Hang C, Tan L, Hu L, Yan Z, et al. The estrogen/miR-338-3p/ADAM17 axis enhances the viability of breast cancer cells via suppressing NK cell’s function. Environ Toxicol. 2023;38(7):1618–27. 10.1002/tox.23791. 37052432 10.1002/tox.23791

[116] Svoronos N, Perales-Puchalt A, Allegrezza MJ, Rutkowski MR, Payne KK, Tesone AJ, et al. Tumor cell-independent estrogen signaling drives disease progression through mobilization of myeloid-derived suppressor cells. Cancer Discov. 2017;7(1):72–85. 10.1158/2159-8290.Cd-16-0502. 27694385 10.1158/2159-8290.CD-16-0502 PMC5222699

[117] Vazquez Rodriguez G, Abrahamsson A, Jensen LD, Dabrosin C. Estradiol promotes breast cancer cell migration via recruitment and activation of neutrophils. Cancer Immunol Res. 2017;5(3):234–47. 10.1158/2326-6066.Cir-16-0150. 28159748 10.1158/2326-6066.CIR-16-0150

[118] Zhang X, Cheng L, Gao C, Chen J, Liao S, Zheng Y, et al. Androgen signaling contributes to sex differences in cancer by inhibiting NF-κB activation in T cells and suppressing antitumor immunity. Cancer Res. 2023;83(6):906–21. 10.1158/0008-5472.Can-22-2405. 36634207 10.1158/0008-5472.CAN-22-2405

[119] Guan X, Polesso F, Wang C, Sehrawat A, Hawkins RM, Murray SE, et al. Androgen receptor activity in T cells limits checkpoint blockade efficacy. Nature. 2022;606(7915):791–6. 10.1038/s41586-022-04522-6. 35322234 10.1038/s41586-022-04522-6 PMC10294141

[120] Kwon H, Schafer JM, Song NJ, Kaneko S, Li A, Xiao T, et al. Androgen conspires with the CD8(+) T cell exhaustion program and contributes to sex bias in cancer. Sci Immunol. 2022;7(73):eabq2630. 10.1126/sciimmunol.abq2630. 35420889 10.1126/sciimmunol.abq2630 PMC9374385

[121] Yang C, Jin J, Yang Y, Sun H, Wu L, Shen M, et al. Androgen receptor-mediated CD8(+) T cell stemness programs drive sex differences in antitumor immunity. Immunity. 2022;55(9):1747. 10.1016/j.immuni.2022.07.016. 36103859 10.1016/j.immuni.2022.07.016

[122] Denton AE, Roberts EW, Fearon DT. Stromal cells in the tumor microenvironment. Adv Exp Med Biol. 2018;1060:99–114. 10.1007/978-3-319-78127-3_6. 30155624 10.1007/978-3-319-78127-3_6

[123] Houthuijzen JM, Jonkers J. Cancer-associated fibroblasts as key regulators of the breast cancer tumor microenvironment. Cancer Metastasis Rev. 2018;37:4577–97. 10.1007/s10555-018-9768-3. 10.1007/s10555-018-9768-3 30465162

[124] Bae WJ, Kim S, Ahn JM, Han JH, Lee D. Estrogen-responsive cancer-associated fibroblasts promote invasive property of gastric cancer in a paracrine manner via CD147 production. FASEB J. 2022;36(11):e22597. 10.1096/fj.202200164RR. 36197688 10.1096/fj.202200164RR

[125] Zhang Y, Cong X, Li Z, Xue Y. Estrogen facilitates gastric cancer cell proliferation and invasion through promoting the secretion of interleukin-6 by cancer-associated fibroblasts. Int Immunopharmacol. 2020;78:105937. 10.1016/j.intimp.2019.105937. 31753587 10.1016/j.intimp.2019.105937

[126] Vivacqua A, Muoio MG, Miglietta AM, Maggiolini M. Differential microRNA landscape triggered by estrogens in cancer associated fibroblasts (CAFs) of primary and metastatic breast tumors. Cancers (Basel). 2019. 10.3390/cancers11030412. 30909585 10.3390/cancers11030412 PMC6468788

[127] Cheng HW, Chen YF, Wong JM, Weng CW, Chen HY, Yu SL, et al. Cancer cells increase endothelial cell tube formation and survival by activating the PI3K/Akt signalling pathway. J Exp Clin Cancer Res. 2017;36(1):27. 10.1186/s13046-017-0495-3. 28173828 10.1186/s13046-017-0495-3 PMC5296960

[128] Zou LB, Shi S, Zhang RJ, Wang TT, Tan YJ, Zhang D, et al. Aquaporin-1 plays a crucial role in estrogen-induced tubulogenesis of vascular endothelial cells. J Clin Endocrinol Metab. 2013;98:4:E672–82. 10.1210/jc.2012-4081. 23450058 10.1210/jc.2012-4081

[129] Conforti F, Pala L, Bagnardi V, De Pas T, Martinetti M, Viale G, et al. Cancer immunotherapy efficacy and patients’ sex: a systematic review and meta-analysis. Lancet Oncol. 2018;19(6):737–46. 10.1016/s1470-2045(18)30261-4. 29778737 10.1016/S1470-2045(18)30261-4

[130] Yang W, Warrington NM, Taylor SJ, Whitmire P, Carrasco E, Singleton KW, et al. Sex differences in GBM revealed by analysis of patient imaging, transcriptome, and survival data. Sci Transl Med. 2019;11:473. 10.1126/scitranslmed.aao5253. 10.1126/scitranslmed.aao5253 PMC6502224 30602536

[131] Lindner AK, Lackner F, Tymoszuk P, Barth DA, Seeber A, Kocher F, et al. Sex hormones influence survival of patients with metastatic urothelial carcinoma undergoing immune checkpoint therapy. Biol Sex Differ. 2023;14(1):38. 10.1186/s13293-023-00522-x. 37277835 10.1186/s13293-023-00522-x PMC10243034

[132] Michmerhuizen AR, Lerner LM, Pesch AM, Ward C, Schwartz R, Wilder-Romans K, et al. Estrogen receptor inhibition mediates radiosensitization of ER-positive breast cancer models. NPJ Breast Cancer. 2022;8(1):31. 10.1038/s41523-022-00397-y. 35273179 10.1038/s41523-022-00397-y PMC8913671

[133] Zhao Y, Wang X, Liu Y, Wang HY, Xiang J. The effects of estrogen on targeted cancer therapy drugs. Pharmacol Res. 2022;177:106131. 10.1016/j.phrs.2022.106131. 35167895 10.1016/j.phrs.2022.106131

[134] Nicolson TJ, Mellor HR, Roberts RR. Gender differences in drug toxicity. Trends Pharmacol Sci. 2010;31(3):108–14. 10.1016/j.tips.2009.12.001. 20117848 10.1016/j.tips.2009.12.001

[135] Wang L, Tu YX, Chen L, Zhang Y, Pan XL, Yang SQ, et al. <article-title update="added">Male‐Biased Gut Microbiome and Metabolites Aggravate Colorectal Cancer Development. Adv Sci. 2023;10(25):e2206238. 10.1002/advs.202206238. 10.1002/advs.202206238 PMC10477899 37400423

[136] Thackray VG. Sex, Microbes, and Polycystic Ovary Syndrome. Trends Endocrinol Metab. 2019;30(1):54–65. 10.1016/j.tem.2018.11.001. 30503354 10.1016/j.tem.2018.11.001 PMC6309599

[137] Pellock SJ, Redinbo MR. Glucuronides in the gut: sugar-driven symbioses between microbe and host. J Biol Chem. 2017;292(21):8569–76. 10.1074/jbc.R116.767434. 28389557 10.1074/jbc.R116.767434 PMC5448086

[138] van Looijer- Langen M, Hotte N, Dieleman LA, Albert E, Mulder C, Madsen KL. Estrogen receptor-β signaling modulates epithelial barrier function. Am J Physiol Gastrointest Liver Physiol. 2011;300(4):621–6. 10.1152/ajpgi.00274.2010. 10.1152/ajpgi.00274.2010 21252046

[139] Menon R, Watson SE, Thomas LN, Allred CD, Dabney A, Azcarate-Peril MA, et al. Diet complexity and estrogen receptor β status affect the composition of the murine intestinal microbiota. Appl Environ Microbiol. 2013;79(18):5763–73. 10.1128/aem.01182-13. 23872567 10.1128/AEM.01182-13 PMC3754184

[140] Elderman M, de Vos P, Faas M. Role of microbiota in sexually dimorphic immunity. Front Immunol. 2018;9:1018. 10.3389/fimmu.2018.01018. 29910797 10.3389/fimmu.2018.01018 PMC5992421

[141] Pernigoni N, Zagato E, Calcinotto A, Troiani M, Mestre RP, Calì B, et al. Commensal bacteria promote endocrine resistance in prostate cancer through androgen biosynthesis. Science. 2021;374(6564):216–24. 10.1126/science.abf8403. 34618582 10.1126/science.abf8403

[142] Calcinotto A, Brevi A, Chesi M, Ferrarese R, Garcia Perez L, Grioni M, et al. Microbiota-driven interleukin-17-producing cells and eosinophils synergize to accelerate multiple myeloma progression. Nat Commun. 2018;9(1):4832. 10.1038/s41467-018-07305-8. 30510245 10.1038/s41467-018-07305-8 PMC6277390

[143] Sivan A, Corrales L, Hubert N, Williams JB, Aquino-Michaels K, Earley ZM, et al. Commensal *Bifidobacterium* promotes antitumor immunity and facilitates anti-PD-L1 efficacy. Science. 2015;350(6264):1084–9. 10.1126/science.aac4255. 26541606 10.1126/science.aac4255 PMC4873287

[144] Viaud S, Saccheri F, Mignot G, Yamazaki T, Daillère R, Hannani D, et al. The intestinal microbiota modulates the anticancer immune effects of cyclophosphamide. Science. 2013;342(6161):971–6. 10.1126/science.1240537. 24264990 10.1126/science.1240537 PMC4048947

[145] Titan AL, He H, Lui N, Liou D, Berry M, Shrager JB, et al. The influence of hormone replacement therapy on lung cancer incidence and mortality. J Thorac Cardiovasc Surg. 2020;159(4):1546–e564. 10.1016/j.jtcvs.2019.10.070. 31866083 10.1016/j.jtcvs.2019.10.070

